# Plasma-Derived Fibrin Hydrogels Containing Graphene Oxide for Infections Treatment

**DOI:** 10.1021/acsmaterialslett.2c01044

**Published:** 2023-03-23

**Authors:** Cristina Martín, Ariadna Bachiller, Juan P. Fernández-Blázquez, Yuta Nishina, José L. Jorcano

**Affiliations:** † Department of Bioengineering, Universidad Carlos III de Madrid, Leganés 28911, Spain; ‡ Institute IMDEA Materials, Getafe 28906, Spain; § Graduate School of Natural Science and Technology, Okayama University, Okayama 700-8530, Japan; # Research Core for Interdisciplinary Sciences, Okayama University, Okayama 700-8530, Japan

## Abstract

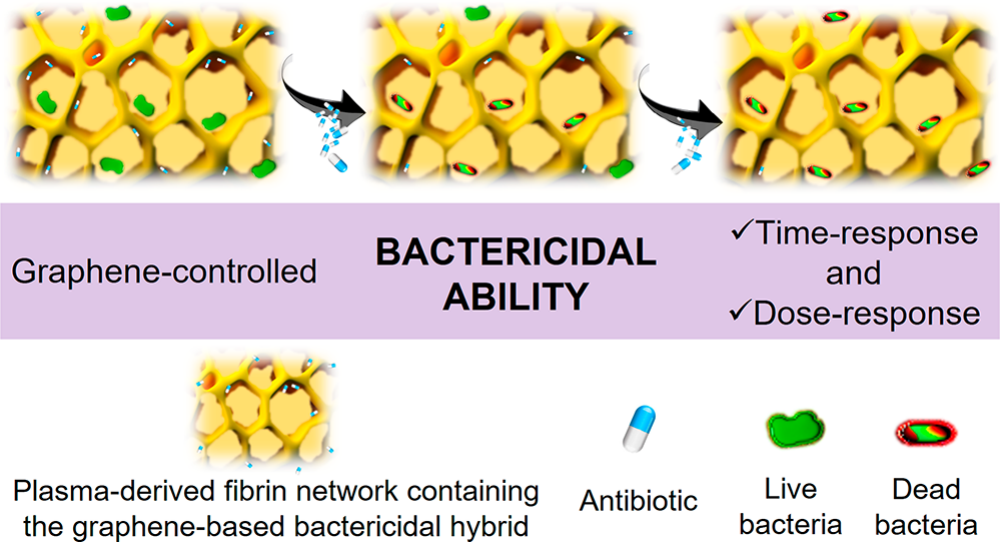

Wound infection is inevitable in most patients suffering from extensive burns or chronic ulcers, and there is an urgent demand for the production of bactericidal dressings to be used as grafts to restore skin functionalities. In this context, the present study explores the fabrication of plasma-derived fibrin hydrogels containing bactericidal hybrids based on graphene oxide (GO). The hydrogels were fully characterized regarding gelation kinetics, mechanical properties, and internal hydrogel structures by disruptive cryo scanning electron microscopies (cryo-SEMs). The gelation kinetic experiments revealed an acceleration of the gel formation when GO was added to the hydrogels in a concentration of up to 0.2 mg/mL. The cryo-SEM studies showed up a decrease of the pore size when GO was added to the network, which agreed with a faster area contraction and a higher compression modulus of the hydrogels that contained GO, pointing out the critical structural role of the nanomaterial. Afterward, to study the bactericidal ability of the gels, GO was used as a carrier, loading streptomycin (STREP) on its surface. The loading content of the drug to form the hybrid (GO/STREP) resulted in 50.2% ± 4.7%, and the presence of the antibiotic was also demonstrated by Raman spectroscopy, Z-potential studies, and thermogravimetric analyses. The fibrin-derived hydrogels containing GO/STREP showed a dose–response behavior according to the bactericidal hybrid concentration and allowed a sustained release of the antibiotic at a programmed rate, leading to drug delivery over a prolonged period of time.

Research in regenerative medicine focuses on the constant seeking and development of new biomaterials, as well as the improvement of existing ones, to provide them with new and adaptable properties to overcome problems related to the repair and regeneration of damaged tissues.^1^

Effective treatments are needed to prevent morbidity and mortality, as well as improvement of the quality of life of patients, regarding skin injuries caused by burns, chronic ulcers, cancer surgery, infections, and other genetic and somatic diseases. The World Health Organization estimates that almost 11 million of new burn injuries per year worldwide require medical attention, with around 180 000 of them leading to death.^2,3^ In particular, wound healing sector has a market value of around $20 billion.

Therefore, there is a clear and urgent demand for the production of skin substitutes and wound dressings to facilitate the healing process and restore skin functionalities avoiding infection. Actually, some challenges still exist in the design and fabrication of integrated systems, particularly for chronic wounds,^4^ presenting significant opportunities for the future development of next-generation smart materials and systems.

In this regard, hydrogels have been considered promising candidates for these particular biomedical applications as they consist of scaffolds with comparable properties to the real tissue ones, such as high water content, ability to encapsulate cells and bioactive molecules and efficient mass transfer, among others.^5,6^ In fact, these soft materials have been widely used for drug delivery, because of their ability to entrap and release different components within their porous matrix.^7,8^ Collagen, elastin, and fibrin-based hydrogels, for instance, have been extensively applied for the synthesis of skin substitutes,^9−13^ as they are the main components of the extracellular matrix (ECM) in the real tissue. In fact, in the skin regeneration context, nanofibers are ideal materials for the treatment and healing of burned or otherwise damaged skin,^6^ since these nanofibers-based networks are attractive materials because of their great cell adhesion.^14^

More specifically, human plasma-derived bilayered skin substitutes can be successfully used in different skin tissue engineering applications, because of their high concentrations of various growth factors.^15,16^ However, there is a constant need for components that are able to improve the mechanical strength and the bioactivity of hydrogels. In that sense, our group has also recently reported how elastin-like recombinamer networks^11^ and thiolated-hyaluronic acid cross-linked with poly(ethylene glycol) diacrylate,^17^ can modify the fiber diameters and compaction of the gel structures and, in consequence, the mechanical properties of the scaffolds. But these polymers are not straightforwardly synthesized and, if commercially available, they are high-priced. In addition, the long experimental time required by three-dimensional (3D) and differentiated bilayer skin equivalents requires additional mechanical stability than that provided by the above-mentioned polymers.

Since the discovery of graphene in 2004,^18^ this material has attracted strong attention due to its unique properties, such as mechanical strength and good stability under chemical and thermal treatments, thus emerging as an interesting new material for a large number of biological and technological applications.^19^

The impact of adding carbon-based materials to the mechanical properties of 3D scaffolds, including hydrogels, is very well-known.^20,21^ In a recent paper, for instance, Tarashi et al. suggested a synergistic effect of reversible interactions induced by bridging of GO sheets between the polymer networks, having also a great contribution to energy dissipation of the hydrogels through desorption of anchored polymer chains, providing, by this way, excellent mechanical properties, significant hysteresis, enhanced toughness recovery, and good fatigue resistance, simultaneously.^22^

On the other hand, Martín et al. have recently summarized the biocompatibility and biodegradability of two-dimensional (2D) materials, including graphene-based derivatives,^23^ highlighting the crucial role of the surface functionalization to control the biodegradability profile, and pointing out the importance of having a good aqueous dispersibility, as it has been extensively reported in several studies.^24−26^ In a different work, Mukherjee et al. studied the degradation of GO by neutrophils, resulting in noncytotoxic and nongenotoxic products.^27^ Additionally, the biomedical benefits provided by graphene-related nanomaterials have been extensively reviewed.^28,29^

Considering the context of this work, maybe the most interesting properties of these graphene derivatives are their possible use as drug delivery systems^30,31^ and antibacterial agents,^32,33^ as different types of nanomaterials have been explored specifically for wound healing properties combined with infection control,^34^ and overall, graphene derivatives constitute a novel approach for the treatment of infection due to their proven bactericidal effect.^35,36^

The above-mentioned properties make graphene-based nanomaterials, such as GO, stand as a safe alternative for the hydrogel’s mechanical enhancement and other bioactive properties of the resulting scaffolds, as recently reported by Pathmanapan et al.,^37^ who emphasized the hypothesis that fibrin hydrogel incorporated with graphene oxide functionalized nanocomposite scaffolds are promising osteoinductive products for bone repair/regeneration. In this regard, the present study explores, to our knowledge, the first ever fabrication of plasma-derived fibrin hydrogels containing bactericidal hybrids based on GO for skin-related applications.

Human plasma-derived hydrogels were synthesized as previously reported by our group (the experimental details can be found in the Supporting Information).^38^ Briefly, plasma aliquots of known fibrinogen concentration were thawed in a water bath at 37 °C. In a typical experiment, to prepare 1 mL of plasma hydrogel, 524.02 μL of plasma (with a fibrinogen concentration of 2.29 mg/mL), 8 μL of Amchafibrin, 387.98 μL of saline (NaCl 0.9% (w/v)), and 80 μL of CaCl_2_ (prepared at 1% w/v) were sequentially added to the vial and mixed.

GO-based nanomaterials were incorporated to the fibrin precursor solutions in order to modify the final structure, properties, and behavior of the resulting hydrogels. To prepare the plasma hydrogels containing the nanomaterial, the volume of NaCl used in the above-mentioned protocol for the preparation of plasma fibrin-derived hydrogels was replaced with the necessary volume of nanomaterial to get the desired filler concentration.

First, the maximum concentration of GO that allows for fibrin polymerization and gel formation was analyzed by timing gelation of fibrin by the flip-flop method. As can be seen in Table 1, the gelation time was accelerated by adding GO up to 0.2 mg/mL, which was chosen as the critical GO concentration for a proper homogeneous gel formation. Above 0.2 mg/mL, the GO seemed to aggregate, showing dispersions consisting of many hydrogel particles (Figure S1 in the Supporting Information). Considering this, only the PLASMA_0, PLASMA_0.1, and PLASMA_0.2 samples were studied further.

**Table 1:** Different Hydrogel Compositions and Gelation Times by the Flip-Flop Method, According to the Concentration of GO

Sample	Concentration of GO, [GO] (mg/mL)	Gelation time (min)	Appearance
PLASMA_0	0	4	homogeneous hydrogel
PLASMA_0.1	0.1	1	homogeneous hydrogel
PLASMA_0.2	0.2	1	homogeneous hydrogel
PLASMA_0.3	0.3	–	dispersion
PLASMA_0.4	0.4	–	dispersion
PLASMA_0.5	0.5	–	dispersion

To better assess the gelation process, the polymerization kinetics was studied by UV spectroscopy, which is a common technique previously performed by our group and others to measure the turbidity resulting from fibrin gelation.^39−41^ The change in the optical density (OD) for the different hydrogels was recorded every 30 s in order to have a closer look at how fibrin polymerization evolves (Figure S2 in the Supporting Information). PLASMA_0 showed an initial phase with no apparent change in absorbance, and the curve reached a plateau after 20 min. However, despite the fact that the opacity of the GO could negatively interfere in the acquisition of a more accurate OD, it is pretty evident that PLASMA_0.1 delayed only around 5 min to reach the plateau, much faster than the control sample without the nanomaterial, and PLASMA_0.2 showed an even more accelerated gelation process.

Finally, in order to better understand the gelation behavior of the hydrogels in the presence of GO, gelation kinetics were analyzed by oscillatory rheological experiments (see Figure 1, as well as Figure S3 in the Supporting Information). The pregel hydrogel solutions (1 mL) were added to the rheometer plate, which had been prewarmed at 37 °C.

**Figure 1:**
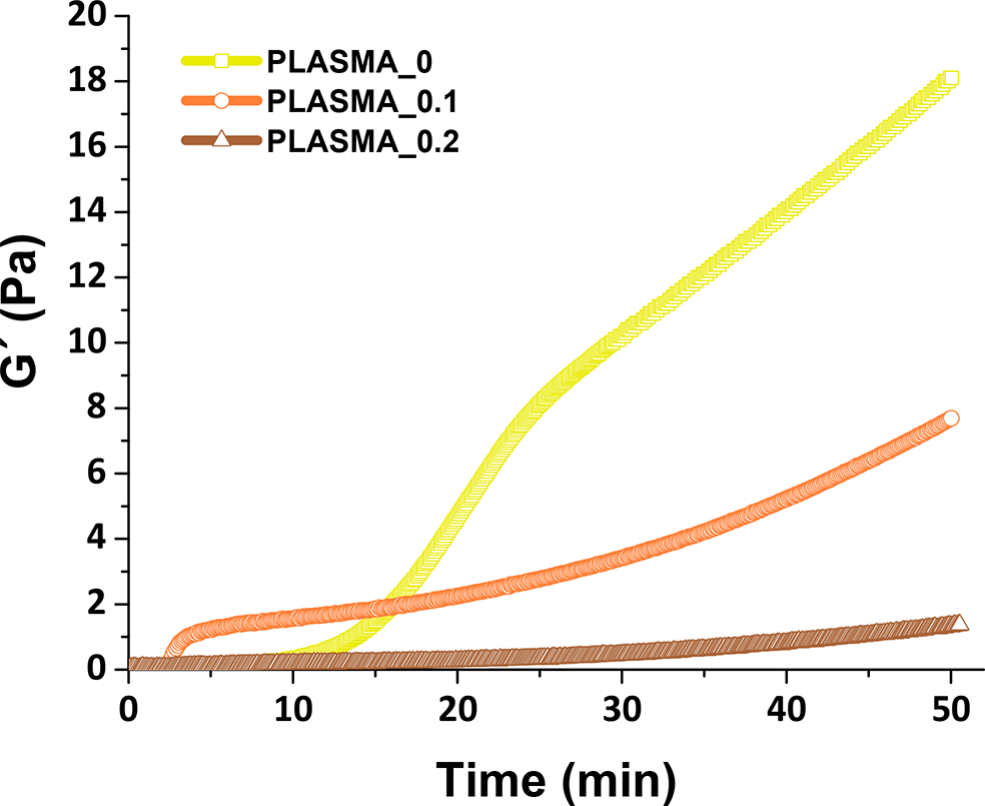
Representative gelation kinetics behaviors via rheological analysis for the PLASMA_0, PLASMA_0.1, and PLASMA_0.2 samples. The experiments were performed in triplicate, but one sample of each hydrogel is represented for simplification.

The storage modulus of PLASMA_0 showed an initial increase in the curve until 8.16 ± 1.87 Pa at ∼25 min, meaning probably a first gelation mechanism. This first rise appeared after only 4 min for PLASMA_0.1; however, the storage modulus at this time point was lower (∼1 Pa) compared to PLASMA_0. This acceleration in the presence of the nanomaterial was also evident for PLASMA_0.2 (see Figure S3 in the Supporting Information). At these time points of the gelation kinetics (25 min for PLASMA_0 and 4 min for PLASMA_0.1 and PLASMA_0.2), the three samples were self-standing hydrogels. Therefore, taking also into account the results obtained from both flip-flop and OD measurement methods, we propose that this first rise in the values of the storage modulus might be produced by the physical changes that occur during the transformation of fibrinogen into fibrin and the fibrin–fibrin interactions. A second slope was also observed in the storage modulus profiles of the three hydrogels, which might be due to the chemical changes driven by transglutaminase Factor XIII, the enzyme responsible of stabilizing the fibrin clot by covalent bonds among fibrin chains,^42^ emphasizing also the dynamic polymerization process over time, in which, after 50 min, the storage modulus of the three samples continues to rise up.

These results point out GO interfering with the fibrin network, acting probably as a physical cross-linker by interacting with proteins and other biomolecules present in a plasma solution, such as fibrinogen, by π–π stacking interactions between GO and aromatic protein residues in addition to hydrophobic interactions^43^ and also with CaCl_2,_^44^ accelerating the creation of self-standing hydrogels. However, since the polymeric fibrin chains could be therefore fixed by this adsorption of GO, the nanomaterial would also slow down the subsequent covalent reaction driven by Factor XIII, highlighting GO also as a “limiting factor” by even preventing fibrin monomers from participating in the polymerization (referred to as “Dispersion” in Table 1 and shown in Figure S1).

Overall, all the gelation time experiments confirmed the acceleration of the gelation process when GO is incorporated into the fibrin-derived pregel solution. This result could be important for the possible 3D bioprinting of the gels. Currently, 3D-bioprinted skin tissues based on human plasma must be supplemented with complex mixtures of polymers (i.e., alginate, methylcellulose, etc.).^45^ The use of low concentrations of GO could provide suitable bioinks with appropriate viscosities and gelation kinetics to produce plasma-derived hydrogels with an accurate pattern in an easy, cost-effective, and controlled way.

The contraction of collagen or fibrin-derived hydrogels is a well-known behavior for these kinds of scaffolds.^38,46^ In order to test the contraction behavior of plasma-derived hydrogels also containing GO, the hydrogels (Vpre-gel = 2 mL) were incubated for 0, 1 h, 3 h, 6 h, 24 h, 48 h, and 7 days in PBS right after gelifying (Figure 2), and the area of the hydrogels was measured at these time points by using eq 1:
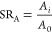
1where SR_A_ is the area swelling ratio, *A_i_* is the area of the hydrogel at each time point *i*, and *A*_0_ is the area of the hydrogel at time zero (*t* = 0, just after extracting the hydrogels from the synthesis vial).

**Figure 2:**
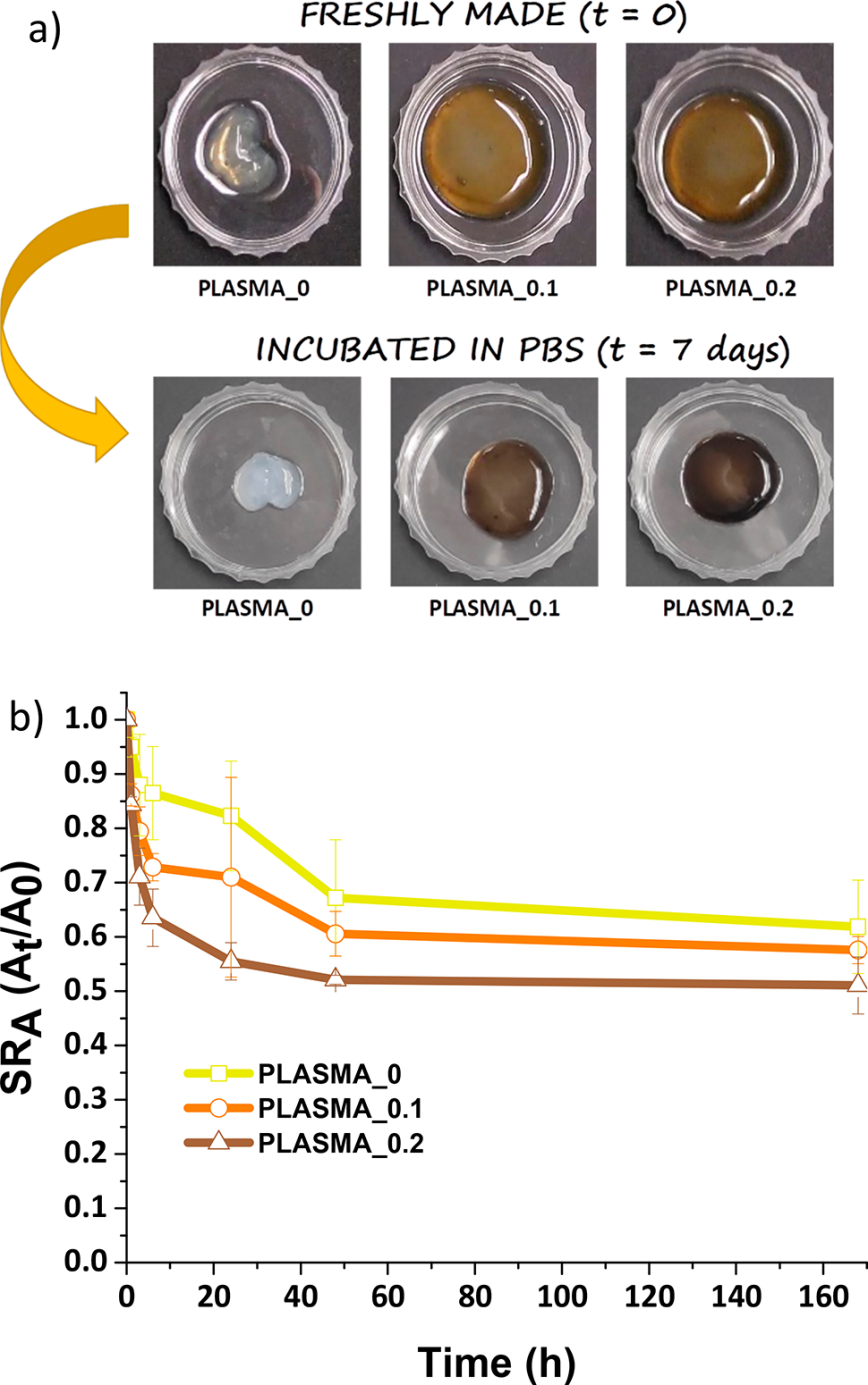
(a) Digital pictures of PLASMA_0, PLASMA_0.1, and PLASMA_0.2 before and after 7 days of incubation in PBS. (b) Area contraction profiles of PLASMA_0, PLASMA_0.1, and PLASMA_0.2.

Figure 2b depicts the area contraction profiles of the three hydrogels. The samples reached the main contraction during the first 25 h of incubation in PBS. After 48 h, the area contraction values remained constant until 7 days. Furthermore, PLASMA_0 contracted less than PLASMA_0.1 and PLASMA_0.2, respectively. This extra area contraction expressed by PLASMA_0.1 and PLASMA_0.2 might be produced by the previously commented interactions of GO with the fibrin network and CaCl_2_. The weight loss produced by the liquid released from the networks when contracted was also analyzed (Figure S4 in the Supporting Information), resulting, as expected, in a main weight loss during the first 25 h of incubation. The sample with less weight loss was PLASMA_0, followed by PLASMA_0.1, and PLASMA_0.2, respectively. The additional cross-linking generated by GO might produce a reduction in the matrix pore size, as discussed below in the morphological characterization by scanning electron microscopy, probably leading to a greater weight loss and area contraction ability, and consequently, to a greater liquid content release. All these studies, taken together, suggest a change in the overall volume of the samples. Interestingly, water molecules in hydrogels behave differently to water in its free state (in bulk) and can be divided into three different conditions: (1) free water, (2) freezable bound water, and (3) nonfreezable bound water.^47^ In this sense, we hypothesize that the interactions of GO with proteins could to some extent prevent the creation of nonfreezable water strongly bound to the polymer, thus influencing afterward the final and total water content of the scaffolds.

To further assess the mechanical properties of the hydrogels, we tested the samples, both the freshly made and incubated in PBS ones, by using TA Instruments Model DMA-Q800 equipment. By this way, we subjected the hydrogels to a uniaxial compression force in order to get the typical stress–strain curves from which the compression moduli can be determined.

The freshly made hydrogels did not show significant differences in their compression moduli (Figure 3a). In the case of the incubated samples (Figure 3b), a rising tendency in the three compression moduli was observed, which was consistent with the increasing content of GO (syneresis was not observed when external pressure was applied on the samples). In addition, the PLASMA_0.2 sample showed a significantly higher compressibility modulus, compared to the same freshly made hydrogel (Figure 3b). These results are consistent with other examples in the literature.^20,48,49^ In this case, when the samples lose their proteins and liquid over time, the role of GO into the fibrin matrix becomes more relevant and the real contribution of the nanomaterial to the properties of the network is clearly revealed.

**Figure 3:**
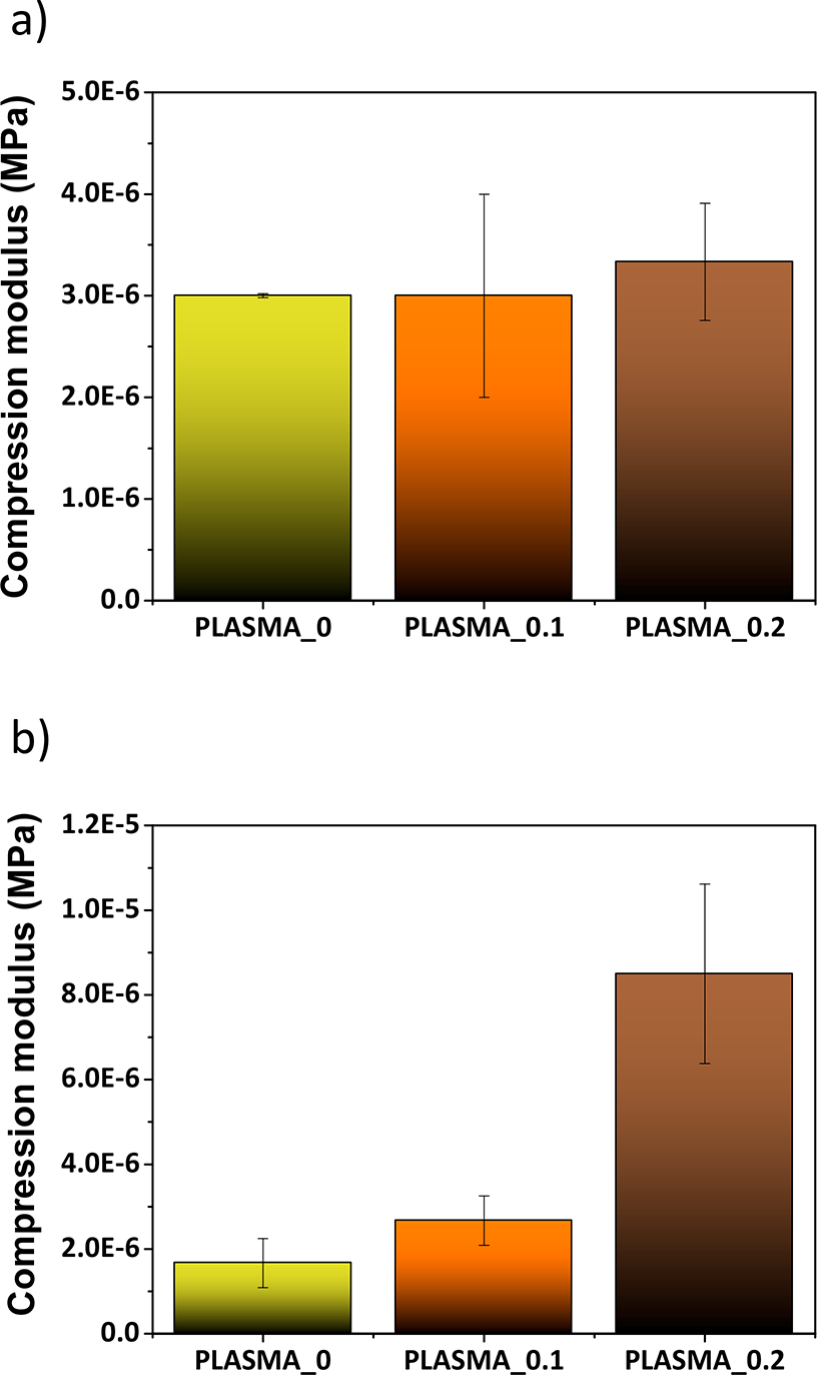
Compression moduli obtained for (a) freshly made and (b) incubated PLASMA_0, PLASMA_0.1, and PLASMA_0.2 hydrogels. The samples were incubated in PBS for three days.

The samples were also characterized by cryo-SEM and cryo-FIB-SEM in order to investigate the structure of the hydrogels. Despite other complementary techniques are widely used to characterize fiber-based hydrogels, such as by supercritical point drying process,^41,50^ both cryo-SEM and cryo-FIB-SEM are highly recommendable novel techniques to characterize biological materials, since both of them allow the hydrogel visualization in its native state, without any previous sample treatment that could modify the network structure and/or composition.^51^

In the case cryo-FIB-SEM,^52^ the technique combines a SEM with a focused ion beam (FIB), by which the electron beam images the cryo-sample surface while the ion beam mills into the surface to expose the interior of the sample generating a trench, and allowing the study of the biological material in 3D (Figure S5 in the Supporting Information) and in a fully hydrated state due to the sample vitrification process. Three different samples were tested: the pregel of PLASMA_0, as well as PLASMA_0 and PLASMA_0.1 after 20 min of polymerization. The time point was chosen based on the previously commented gelation kinetic results, and the sample preparation was optimized avoiding the drying of the gel solution during the gel formation in 200-μm-deep, 6-mm-diameter planchettes (Figure S6 in the Supporting Information). Fibrin chains were easily identified on the trenches of the hydrogels (PLASMA_0 and PLASMA_0.1) after 20 min of gelation (Figure 4), contrary to the pregel solution of PLASMA_0, in which no fibers can be clearly identified since the pregel is vitrified at *t* = 0, before fibrin polymerization starts (Figure S7 in the Supporting Information). Furthermore, the pattern drawn by fibrin fibers is more evident for the PLASMA_0.1 sample, probably due to the higher contrast provided by GO, which is integrated into the fibrin network.

**Figure 4:**
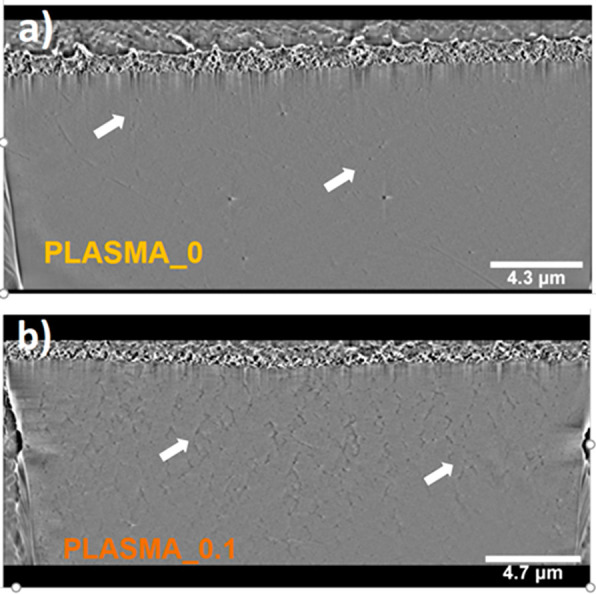
Cryo-FIB-SEM images of (a) PLASMA_0 (scale bar = 4.3 μm) and (b) PLASMA_0.1 (scale bar = 4.7 μm) after 20 min of polymerization. White arrows indicate some example fibrin chains.

On the other hand, cryo-SEM enables the observation of bulk biological materials in hydrated conditions by conversion of liquid water to solid by cryofixation. Therefore, it has been used not only for observation of the ultrastructure in biological materials but also for observation of water distribution within tissues.^53^

Cryo-SEM was used here to validate possible alterations on the structure morphologies of the hydrogels, not only in the freshly made ones, but also in the scaffolds that had been incubated in PBS for 3 days, and in the presence (or absence) of GO. This technique evidenced structural differences between the PLASMA_0, PLASMA_0.1, and PLASMA_0.2 samples: the hydrogels presented a honeycomb structure that suffered changes as the concentration of GO was increased. The length of the pores was progressively reduced with the increase of the nanomaterial concentration for freshly made PLASMA_0.1 and PLASMA_0.2 samples, compared to the PLASMA_0 sample (see Figures 5c and 5e, Figures 6c and 6e, and Figures 7c and 7e). This behavior is in agreement with other hydrogels containing graphene derivatives published previously in the literature.^54,55^ Focusing on incubated hydrogels, the length of the pores slightly decreased, compared to the corresponding sizes of the freshly made materials (see Figures 5b, 5d, and 5f, Figures 6b, 6d, and 6f, Figures 7b, 7d, and 7f). This result correlates with the area contraction and weight loss abilities discussed above. It is important to highlight the fibrillary morphologies of the hydrogels resulting from the fibrin chains, which can be easily observed in Figures 5b, 6b, and 7b (this was not the case when we analyzed the pore walls of the freshly made hydrogels (see Figure S8 in the Supporting Information). Additionally, while PLASMA_0 had a clear network of fibrin (Figure 5b), PLASMA_0.1 showed a more irregular and denser structure (Figures 6b and 6d). This alteration of the honeycomb-like pattern and the denser appearance of the fibrin-based walls was emphasized for PLASMA_0.2 (Figures 7b and 7d). All these results point out and confirm the interaction of GO with fibrin polymer chains, and therefore, the structural role of the nanomaterial on the fibrin network.

**Figure 5:**
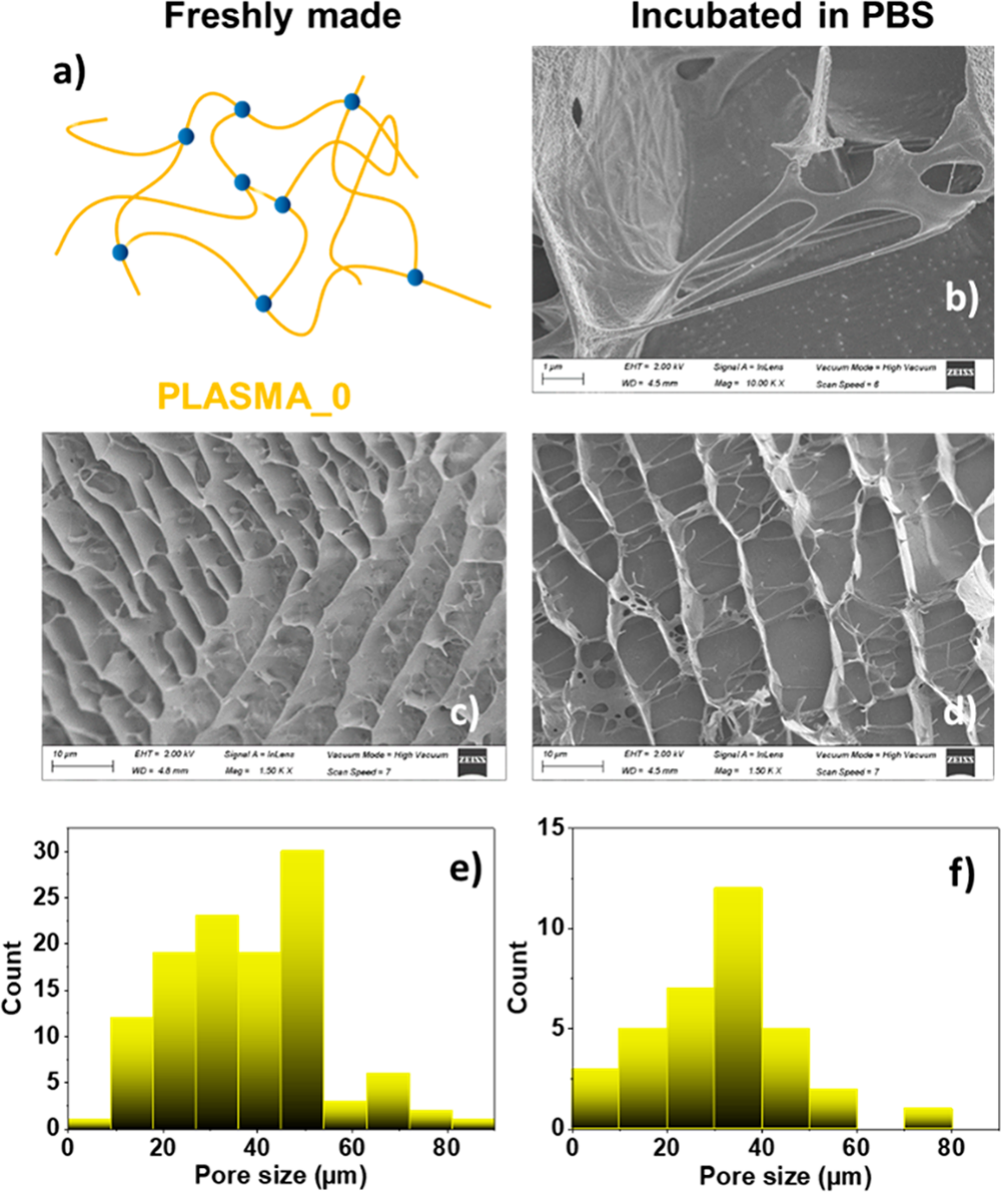
Cryo-SEM characterization of PLASMA_0. (a) Schematic representation of the hydrogel. (b–d) Representative cryo-SEM images of the pore size and morphologies corresponding to the freshly made material (panel (c), scale bar = 10 μm) and that incubated in PBS (panel (b), scale bar = 1 μm; panel (d), scale bar = 10 μm). Pore size distributions of (e) the freshly made material and (f) that incubated in PBS for three days.

**Figure 6:**
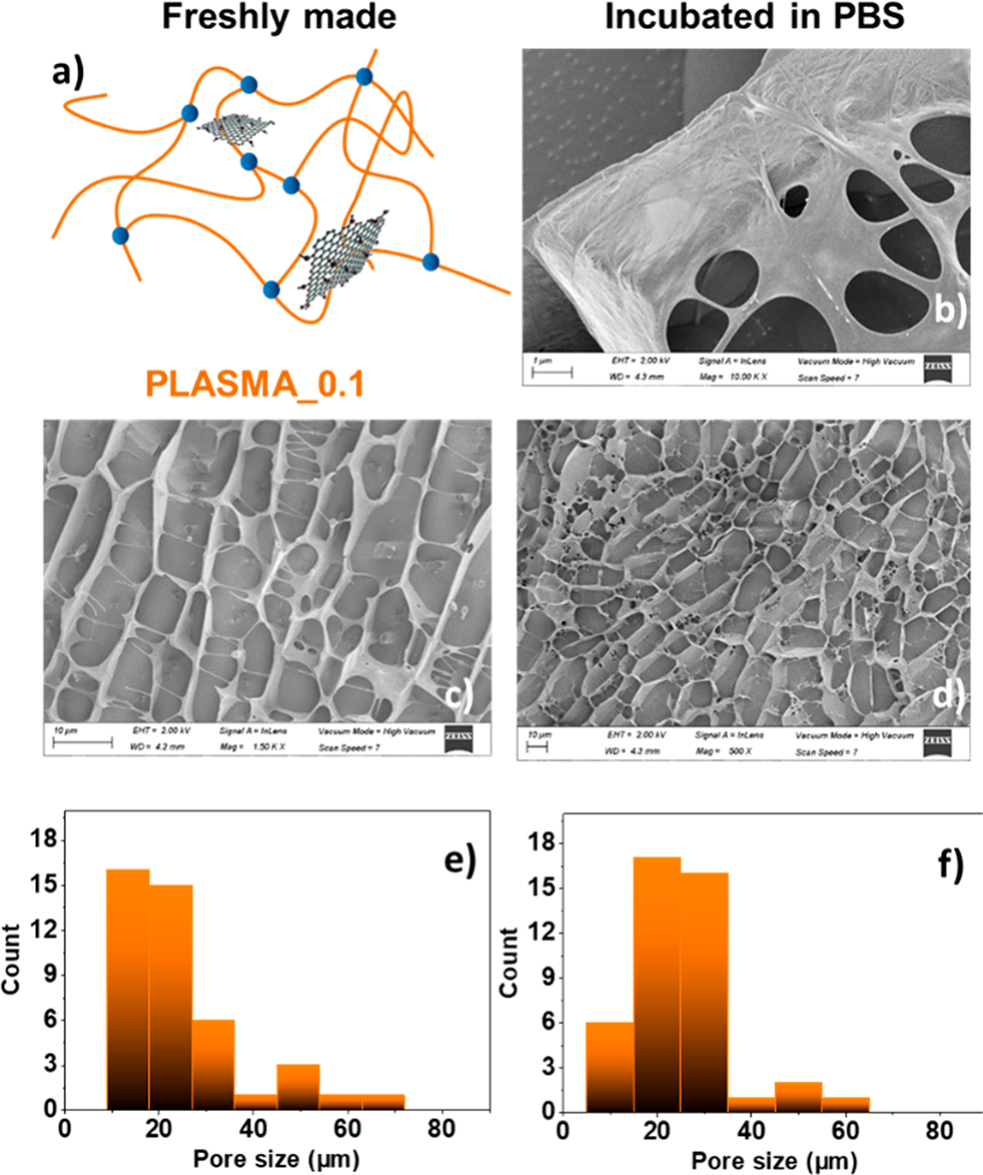
Cryo-SEM characterization of PLASMA_0.1. (a) Schematic representation of the hydrogel. (b–d) Representative cryo-SEM images of the pore size and morphologies corresponding to the freshly made material (panel (c), scale bar = 10 μm) and that incubated in PBS (panel (b), scale bar = 1 μm; panel (d), scale bar = 10 μm). Pore size distributions of (e) the freshly made material and (f) that incubated in PBS for three days.

**Figure 7:**
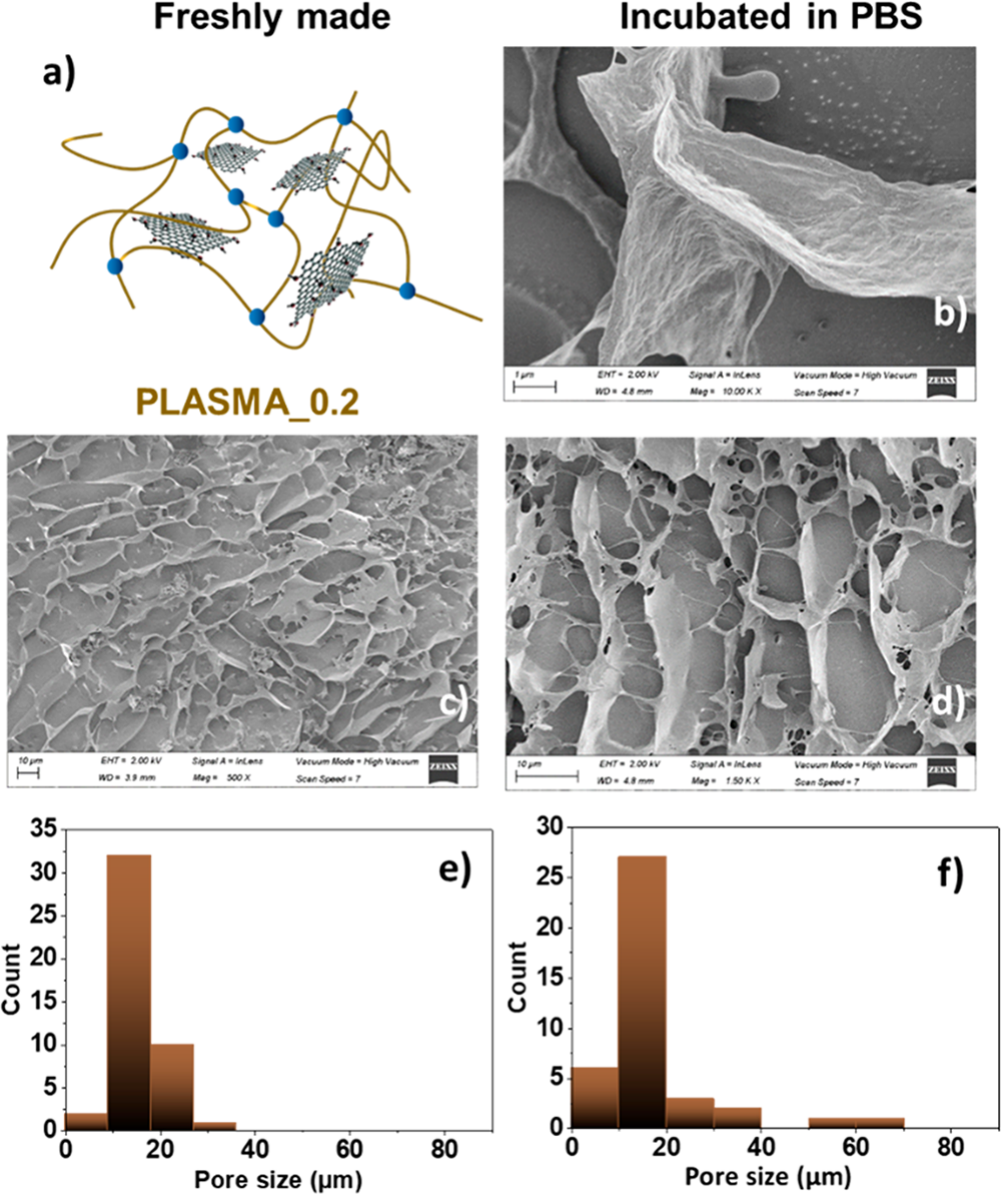
Cryo-SEM characterization of PLASMA_0.2. (a) Schematic representation of the hydrogel. (b–d) Representative cryo-SEM images of the pore size and morphologies corresponding to the freshly made material (panel (c), scale bar = 10 μm) and that incubated in PBS (panel (b), scale bar = 1 μm; panel (d), scale bar = 10 μm). Pore size distributions of (e) the freshly made material and (f) that incubated in PBS for three days.

As a proof of concept, the bactericidal abilities of these plasma-derived hydrogels containing graphene-related materials were tested. Different mechanisms concerning the bactericidal effect of graphene-based nanomaterials have been proposed in the literature,^33,56−58^ which can be summarized in (i) nanoblade effect (membrane cutting and leakage of intracellular components), (ii) wrapping (bacteria wrapped by nanosheets), and (iii) oxidative stress induction (membrane damage via generation of reactive oxygen species). However, as already discussed in the previous sections, GO remains inside the plasma-derived hydrogels, most probably due to its interaction with fibrin polymer chains and its role as a cross-linker agent taking part of the hydrogel structure. Because of this reason, and due to the anchorage of GO to the fibrin network, only the mechanism related to the induction of oxidative stress could be expected. However, no bactericidal effect was observed at the concentrations used (i.e., 0.1 and 0.2 mg/mL), neither of the GO dispersion nor of the GO-containing hydrogels. In fact, concerning the GO dispersion experiments, it was necessary to increase the concentration of nanomaterial to 2 mg/mL to observe a 20% decrease in the bacteria viability compared to the culture non treated with GO, but only with PBS (control). Based on these observations, and in order to obtain the desired bactericidal effect from the plasma-derived hydrogels, GO was functionalized with streptomycin (GO/STREP), one of the few antibiotics effective against multiresistant bacteria,^59^ and then used as bactericial hybrid material.

The synthesis of GO/STREP hybrid was achieved in an easy and single-step process (Figure S9 in the Supporting Information). Briefly, the nanomaterial was mixed with the drug in aqueous solution, and the mixture was stirred at 4 °C overnight. After purification, the loading content of the drug resulted in 50.2% ± 4.7% (see the Supporting Information for details). The adsorption mechanism is most probably based on supramolecular interactions between the drug and GO (van der Waals, hydrogen bonding, aromatic stacking, π–π interactions, etc.) and electrostatic and charge transfer processes, as previously reported in the literature.^60,61^

In order to ascertain the presence of the drug on the GO platform, Z-potential measurements were performed. The values obtained were −35.63 ± 1.36 mV and −1.34 ± 1.36 mV for GO and GO/STREP, respectively. As expected, the presence of the drug led to an increase in the GO-based hybrid surface charge, due to the intrinsic positive charge of streptomycin.^62^ Additionally, the surface modification of GO when functionalized with streptomycin was also confirmed by Raman spectroscopy. As it can be observed in Figure 8a, both the GO and the GO/STREP Raman spectra show the typical peaks for these types of carbon-based materials, meaning the so-called “D and G bands”, corresponding to disordered carbon and to the sp^2^ tangential mode, respectively. The D band appears at ∼1350 cm^–1^ and the G band arises at ∼1600 cm^–1^.^63^ The increase in the *I*_D_/*I*_G_ value of GO/STREP (with *I*_D_/*I*_G_ = 1.02) compared to GO (with *I*_D_/*I*_G_ = 0.91) could be due to a larger density of structural defects on GO after mixing with the drug,^64−66^ indicating the deformation of ordered structure and suggesting the appearance of different groups due to the presence of the drug adsorbed on the GO surface. Finally, the thermogravimetric analysis (TGA) curve of GO/STREP displayed a percentage residual weight higher than that of GO at 600 °C, presenting weight losses at similar temperatures to STREP alone (control), and confirming, therefore, the drug loading of STREP onto the GO surface (Figure 8b).

**Figure 8:**
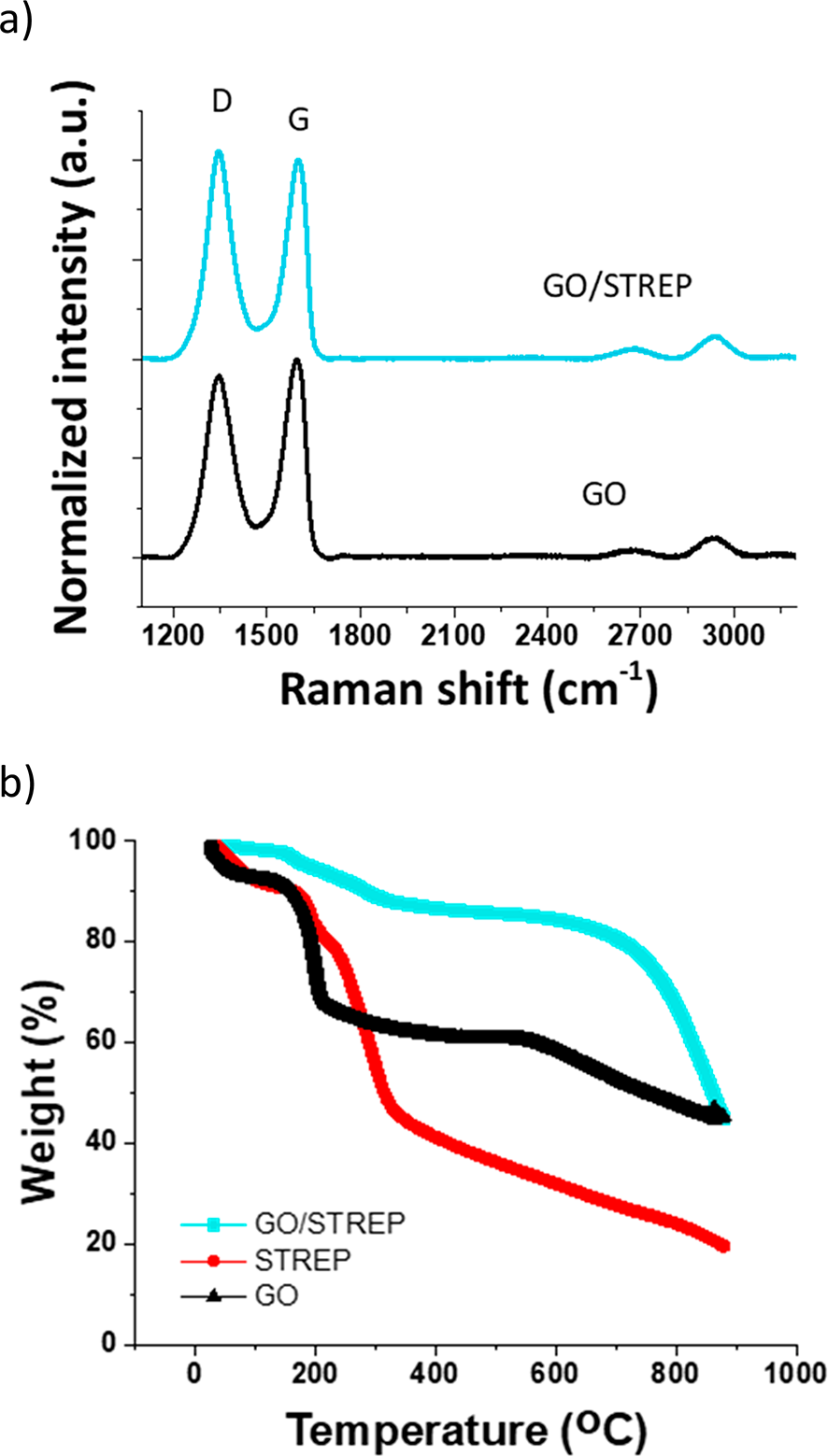
(a) Average Raman spectra for GO (bottom) and GO/STREP (top) materials. (b) Representative TGA curves of the freeze-dried dispersions of GO, STREP, and GO/STREP materials.

Human plasma-derived hydrogels were synthesized as previously described, using GO/STREP hybrid instead of GO alone, at two different concentrations: 0.1 mg/mL (PLASMA_0.1_GO/STREP) and 0.2 mg/mL (PLASMA_0.2_GO/STREP). As could be predicted, the gelation time, the internal structure, and the pore size of the resulting hydrogels were dependent on the GO concentrations, showing similar results to the already commented above regarding the morphological characterization by scanning electron microscopy when using GO without carrying the drug (see Figure S10 in the Supporting Information).

In order to quantify the bactericidal ability of our hydrogels containing (or not) the GO/STREP hybrid, we performed the colony counting method. Briefly, *Escherichia coli* (*E. coli*) were incubated with the different freshly prepared hydrogel samples (pregel volumes of 300 μL) in PBS at 37 °C under a shaking speed of 210 rpm for 1 or 2 h. Aliquots of samples were withdrawn, diluted with PBS and then spread onto LB agar plates. After incubation at 37 °C, the capacity of the bacteria to form colonies was measured by counting the number of colony-forming units (see the experimental methods and Figure S11 in the Supporting Information for more details). The bactericidal activity of five different hydrogels (Figure 9) was tested. The hydrogel derived from human plasma in the absence of the hybrid (PLASMA_0) was used as a control sample. In fact, the number of colonies after treatment with PLASMA_0 was equal to that of the *E. coli* control plate without any hydrogel treatment (data not shown), since, as expected, PLASMA_0 has no bactericidal capacity. Moreover, two additional control hydrogels were prepared: PLASMA_0.1_STREP and PLASMA_0.2_STREP. The loading content of 50.2% ± 4.7% calculated after the synthesis and characterization of GO/STREP, was used to ensure that both samples contained the same drug amount as PLASMA_0.1_GO/STREP and PLASMA_0.2_GO/STREP, respectively; however, in the absence of GO, the antibiotic was freely dispersed into the pores of both hydrogels.

**Figure 9:**
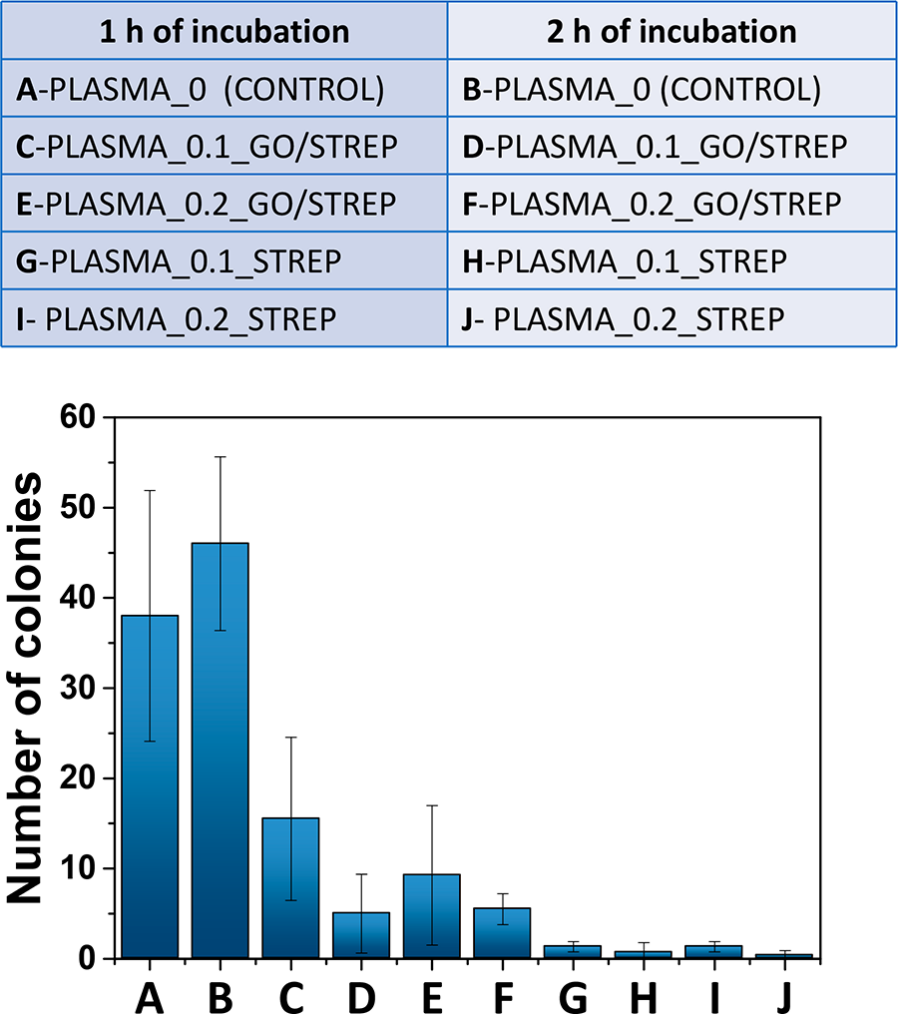
Letter-sample correlations and average number of colonies counted for the different hydrogel samples tested against *E. coli* after 1 h of incubation (A, C, E, G, and I) and 2 h of incubation (B, D, F, H, and J).

As can be observed in Figure 9, a decrease in the number of colonies was obtained when bacteria were incubated in the presence of PLASMA_0.1_GO/STREP or PLASMA_0.2_GO/STREP (C–F in Figure 9), compared to PLASMA_0 (A and B, respectively, in Figure 9), thanks to the presence of the bactericidal GO/STREP hybrid. A dose-dependent response was obtained, especially after 1 h of incubation with *E. coli*, according to the hybrid concentration added to the hydrogel: about half of colonies were counted when using PLASMA_0.2_GO/STREP (E in Figure 9), compared to PLASMA_0.1_GO/STREP (C in Figure 9). Additionally, a time-dependent behavior was also observed when treating *E. coli* with these two hydrogels, as one can note that the number of colonies after 2 h of incubation is lower, in both cases, compared to 1 h (D vs C and F vs E in Figure 9). Finally, it is important to note that a controlled release behavior of streptomycin does not happen when using the drug freely dispersed into the pores of the hydrogels (G–J in Figure 9). This result is not surprising, considering that, in PLASMA_0.1_STREP and PLASMA_0.2_STREP hydrogels, the antibiotic is not adsorbed onto GO (as it is in the PLASMA_0.1_GO/STREP or PLASMA_0.2_GO/STREP samples), resulting in a sudden and uncontrolled release of all the drug over time and contrary to what occurs using the hybrid, with which a sustained release is achieved thanks to the antibiotic desorption process (Figure S12 in the Supporting Information). Representative images of the agar plates with *E. coli* colonies that resulted from the different hydrogel samples tested against bacteria after 1 h (A, C, and E in Figure 9) and 2 h (B, D, and F in Figure 9) of incubation are displayed in Figure S13 in the Supporting Information.

Considering the possible future application of plasma-derived hydrogels containing GO-based bactericidal hybrids as skin dressings in wound treatment, the cell viability of human fibroblast cells (hFBs), which are the main cells in the dermis layer of skin, was studied when exposed to both the GO/STREP dispersions and plasma-derived hydrogels.

First, GO/STREP dispersions at 0.1 and 0.2 mg/mL were tested using LDH assay. Figure 10A shows the cytotoxicity of the bactericidal hybrid dispersion at both concentrations, showing no decrease in viability of hFBs in both cases, compared to the positive control (hFBs that were not exposed to bactericidal GO/STREP nanomaterial). As expected, the GO dispersion in the absence of the drug also showed no decrease in hFBs viability (data not shown). Furthermore, evident differences were not observed when comparing GO/STREP dispersions at 0.1 and 0.2 mg/mL. Therefore, only PLASMA_0 and PLASMA_0.2_GO/STREP hydrogels were studied, as the latter contains the highest concentration of bactericidal hybrid for hydrogel formation. As shown in Figure 10B, the viability of hFBs did not decrease when the cells were incubated in the presence of both hydrogel samples. In fact, a slight increase in cell viability was observed in both cases, compared to the positive control (hFBs that were not exposed to any hydrogel), which could be explained by the unique composition of the plasma,^67^ which includes plasma proteins, platelets, enzymes, and growth factors, which can promote cell proliferation.^68^ In this regard, it is also worth mentioning the importance of using human blood plasma as a source of human fibrin for autologous skin replacement, given its potential to embed fibroblasts to produce natural collagen.^69^Figure S14 in the Supporting Information shows hFBs embedded in our plasma-derived fibrin hydrogels containing bactericidal hybrids (i.e., PLASMA_0.2_GO/STREP).

**Figure 10:**
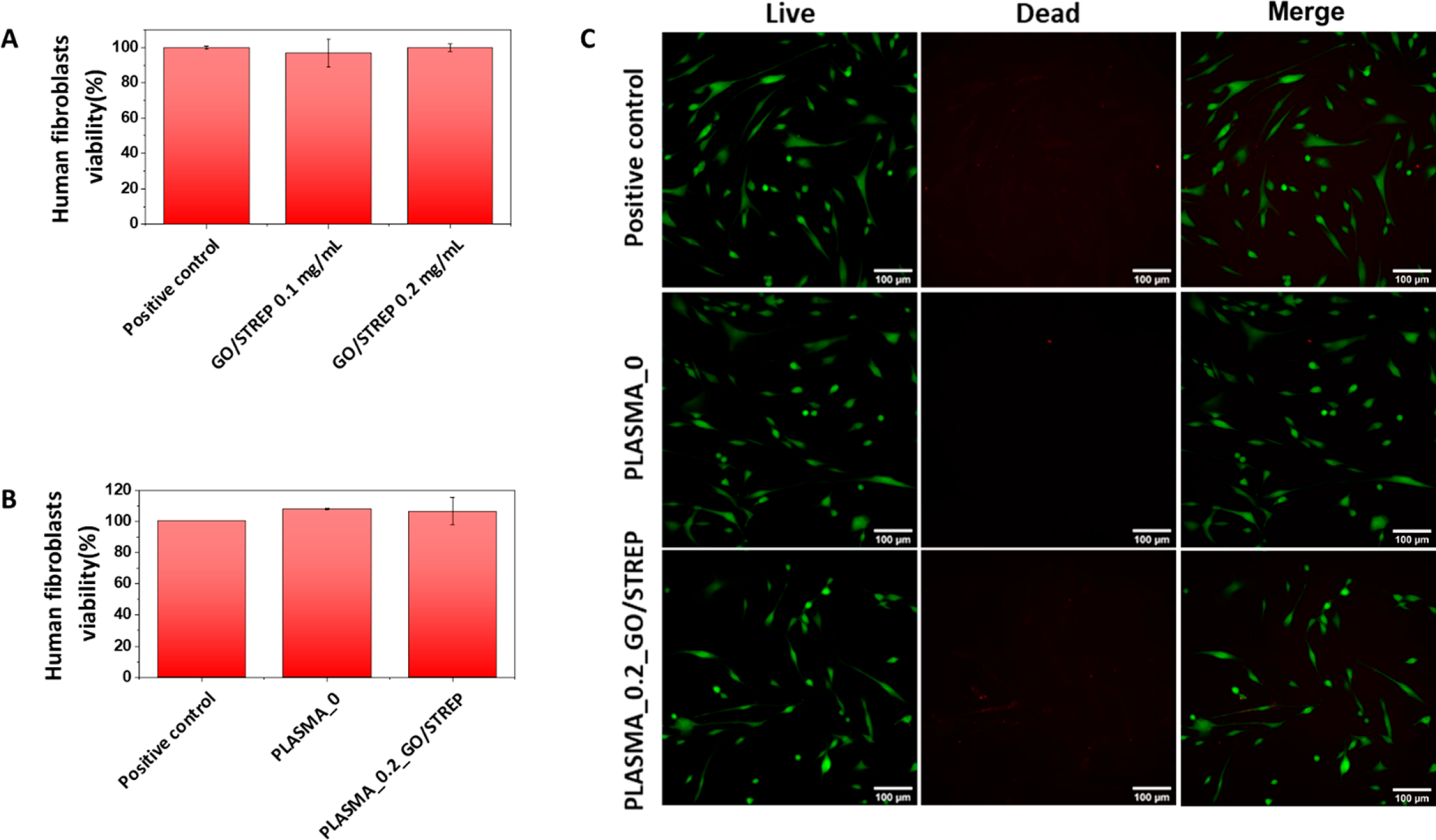
(A) Human fibroblast viability after 24 h of incubation in the presence of GO/STREP at 0.1 and 0.2 mg/mL, determined by LDH assay. (B) Human fibroblast viability after 24 h of incubation in the presence of PLASMA_0 and PLASMA_0.2_GO/STREP hydrogels, determined by LDH assay. (C) Human fibroblast viability after 24 h of incubation in the presence of PLASMA_0 and PLASMA_0.2_GO/STREP hydrogels, determined by live/dead assay (scale bars = 100 μm). The positive control sample consisted of hFBs that were not exposed to any nanomaterial dispersion or hydrogel.

Cell viability of hFBs was also characterized using Live/Dead assay for mammalian cells. As described above, no differences were observed when comparing GO/STREP dispersions at 0.1 and 0.2 mg/mL when testing LDH activity. Therefore, only PLASMA_0 and PLASMA_0.2_GO/STREP hydrogels were studied using live/dead assay.

As expected, the cell viability of PLASMA_0 and PLASMA_0.2_GO/STREP hydrogels was comparable to that of the positive control, as almost no dead cells were found in any case (Figure 10C) (the live/dead results obtained from the negative control sample, consisting of hFB that had been exposed to 10% of DMSO for 24 h, are shown in Figure S15 in the Supporting Information). These results are in agreement with the above-commented ones concerning LDH-based studies, and emphasize once more the great potential of our plasma-derived fibrin hydrogels containing GO-based bactericidal hybrids in relation to the fabrication of bactericidal skin dressings for wound healing and/or in-vitro-produced skin equivalents for grafting (for instance, for extensively burned patients).^15,70,71^

This work explored the fabrication of plasma-derived fibrin hydrogels containing GO-based bactericidal hybrids. The GO-containing hydrogels were fully characterized, which allowed us to confirm and point out the critical structural role of the nanomaterial in the plasma-derived network. Afterward, GO was used as a carrier, loading streptomycin on its surface, to achieve the bactericidal hybrid GO/STREP, which presented a loading content for the drug of 50.2% ± 4.7%. The fibrin-derived hydrogels containing GO/STREP were not cytotoxic to human fibroblasts, and they showed a dose response as a function of the concentration of the bactericidal hybrid against *E. coli*. A sustained release of the antibiotic was also achieved, leading to a drug delivery capacity of the hydrogels over an extended period of time.

Overall, these results emphasize the great potential of our plasma-derived fibrin hydrogels containing bactericidal GO hybrids regarding the fabrication of bactericidal skin dressings for wound healing and/or in-vitro-produced skin equivalents for grafting (for instance, for extensively burned patients). In recent years, several preclinical trials have been initiated to investigate the safety and efficacy of GO-based therapies for cancer, or neurological disorders, but rigorous testing and evaluation are necessary to ensure that these materials are safe and effective for human use. Further in vitro and in vivo experiments with *Pseudomonas aeruginosa*, which is one of the most abundant multidrug-resistant Gram-negative bacteria in hospitals, are needed to assess the applicability of these skin-inspired scaffolds, but it is also important to highlight the wider utility that our hydrogels could have for other tissue engineering applications, such as the industrial testing of drugs and cosmetic products.

## Supporting Information Available

Materials, experimental details, and supporting figures, including gelation kinetics determined by changes in the optical density, digital pictures of gel mixtures containing above 0.2 mg/mL of GO (“heterogeneous dispersions”), representative gelation kinetics behaviors via rheological analysis in a log scale, weight loss profiles, cryo-FIB-SEM image of pregel solution, representative Cryo-SEM images of the pore-size and morphologies corresponding to the freshly made materials ((a) PLASMA_0, (b) PLASMA_0.1, and (c) PLASMA_0.2), TGA characterization of GO/STREP, scheme of the bactericidal experiments, streptomycin release kinetics for PLASMA_0.1_GO/STREP and PLASMA_0.2_GO/STREP hydrogels, and representative images of the agar plates with *E. coli* colonies that resulted from the different hydrogel samples tested against bacteria, cryo-SEM images of PLASMA_0.1_GO/STREP and PLASMA_0.2_GO/STREP hydrogels with and without embedded human fibroblasts, and negative control for live/dead experiments (PDF)
