# Supplementary material for: Plasma-Derived Fibrin Hydrogels Containing Graphene Oxide for Infections Treatment
Source: ACS Mater Lett. 2023 Mar 23;5(4):1245–55. doi: 10.1021/acsmaterialslett.2c01044 (PMC10842975; doi:10.1021/acsmaterialslett.2c01044)
Supplement: Supplementary file 1 — tz2c01044_si_001.pdf [file tz2c01044_si_001.pdf]

Supporting Information

Plasma-derived fibrin hydrogels containing graphene oxide for infections treatment

Cristina Martín[#,‡,*], Ariadna Bachiller[#,‡], Juan P. Fernández-Blázquez[§], Yuta Nishina[¥,ƍ], José L. Jorcano[#,*].

[#] Department of Bioengineering & Aerospace Engineering, Universidad Carlos III de Madrid, Leganés, Spain.

§ Institute IMDEA Materials, Getafe, Spain.

¥ Graduate School of Natural Science and Technology, Okayama University, Okayama, Japan.

ƍResearch Core for Interdisciplinary Sciences, Okayama University, Okayama, Japan.

[‡] There authors contributed equally to this work.

1. EXPERIMENTAL METHODS

1.1. Materials

Human platelet-poor plasma was obtained from a blood bank (Banco de Sangre del Centro Comunitario de Transfusión del Principado de Asturias (CCST)), stored at -80ºC upon arrival to the facility and, finally, defrosted prior to its use. Graphene oxide (GO) was provided by Prof. Yuta Nishina (NiSiNa Materials, Co. Ltd., Okayama University, Japan).[1-3] Streptomycin sulphate (STREP) was obtained from Merck. Amchafibrin was provided by Rottafarm, Spain. Calcium chloride ($CaCl_2$), sodium chloride (NaCl), phosphate buffer saline (PBS) purchased from Sigma Aldrich, USA. Human fibroblast cells (hFBs) were obtained from ThermoFisher Scientific. The culture medium was Bulbecco's modified Eagle's medium (DMEM, BiochromKG) containing 10% fetal bovine serum (FBS) and 1% Penicilin/Streptomycin. Luria Broth Base (Miller's LB Broth Base) ™ and LB agar, Miller were purchased from Sigma Aldrich, USA, and Fisher Scientific, respectively. *Escherichia coli* (lyophilized cells) were purchased from Merck. A Cytotoxic 96@ Non-Radioactive kit from Promega was used for the LDH assays. Cell viability was also studied using Live/Dead® Viability assay (Thermo Fisher Scientific, USA).

1. 2. Synthesis and characterization of fibrin-derived hydrogels containing GO.

Human plasma-derived hydrogels were synthesized as previously reported by our group.[4] Briefly, plasma aliquots of known fibrinogen concentration were thawed in a water bath at 37°C. In a typical experiment, to prepare 1mL of plasma hydrogel, 524.02 µL of plasma (with a fibrinogen concentration of 2.29 mg/mL), 8 µl of Amchafibrin, 387.98 µL of saline (NaCl 0.9 % (w/v)) and 80 µL of $CaCl_2$ (prepared at 1% w/v) were sequentially added to the vial and mixed. The final concentrations of fibrinogen and $CaCl_2$ in the plasma hydrogels were adjusted to 0.12% and 0.08% (w/v), respectively, by conveniently varying the plasma and saline volumes used.

To prepare the plasma hydrogels containing any nanomaterial, the volume of NaCl used in the protocol for the preparation of plasma fibrin-derived hydrogels was replaced with the necessary volume of nanomaterial to get the desired filler concentration. For instance, considering an initial dispersion of GO of 7.5 mg/mL, to prepare 1mL of PLASMA_0.1, 524.02 µL of plasma (with a fibrinogen concentration of 2.29 mg/mL), 8 µl of Amchafibrin, 374.65 µL of saline (NaCl 0.9 % (w/v)), 13.33 µL of GO dispersion and 80 µL of $CaCl_2$ (prepared at 1% w/v) were sequentially added to the vial and mixed.

The volumes of the hydrogels necessarily varied depending on the characterization technique, but the specific ratio of the components, was always constant. All hydrogels were incubated for 1 h at 37 °C and 5% CO_2 for complete gelation. The experiments described in this article were performed using plasma from the same batch.

1.2.1. Gelation time and kinetics:

Three different methods were used to determine the gelation time of the hydrogels: the flip-flop test, the turbidimetric analysis by UV measurements, and the oscilatory rheological studies.

1.2.1.1. Flip-flop test

This test is based on the free flowing of liquid inside the crystal vials where the hydrogels were synthesized.[5] Basically, the hydrogel solutions were prepared and incubated at 37°C. Vials were then tilted every 1 min. When there was no liquid left in the vial and the hydrogel remained stuck at the bottom, this was considered as the gelation time.

1.2.1.2. Gelation kinetics via turbidimetric analysis

To further study the gelation kinetics of the hydrogels, a SynergyTM HTX Multi-Mode Microplate Reader (Winooski, VT, USA) was used to measure turbidity.[6-8] First, we determined the most adequate wavelength to study the sol-gel transition. To this end, the reader made a sweep from 200 nm to 800 nm, in 5 seconds time intervals, at 37°C. Two rows of 300 µL samples were introduced inside a 96-well microplate.

After this first wavelength sweep, the most adequate wavelength was chosen and the samples were introduced in the microplate reader to measure the hydrogel (300 µL) absorbance in the microplate reader for 2 h, at 37 °C, in 30 s intervals, at 340 nm.

1.2.1.3. Gelation kinetics via rheological analysis

The oscillatory rheological characterization of PLASMA_0, PLASMA_0.1 and PLASMA_0.2 was performed in a TA Instruments AR-G2 Rheometer (New Castle, USA) using a sand-blasted aluminium parallel plate of 40 mm diameter and a Peltier device for temperature control. 1 mL of pre-gel plasma-derived fibrin hydrogel dispersion was added between the plates at 37°C, and the gap was closed until the liquid reached the correct geometry. Then,

the temperature of the plate was kept constant at 37°C, the strain was set at 1.0% and the frequency at 1.0 Hz for a period of 1h in order to record all the changes that occurred during gelation. From this measurement, the storage modulus (G') was recorded as a time function by an oscillatory time sweep.[9,10]

1.2.2. Area contraction and weight loss experiments:

The area contraction of the hydrogels was determined by measuring the reduction in surface area.[4,11] PLASMA_0, PLASMA_0.1 and PLASMA_0.2 (2 mL of final volume) were prepared in glass vials. Once the gelation reaction had taken place, the hydrogels were detached using 1 mL of warned phosphate-buffered saline (PBS) at 37ºC and transferred to pre-weighted p35 Petri dishes. The hydrogels were submerged in 3 mL of PBS (replenished after each measurement) and incubated at 37°C, 5% CO_2 and 40% relative humidity. Area contraction was measured at time points of 0h, 1h, 3h, 6h, 24h, 48h and 7 days. The hydrogels were photographed over a black background using a 30 cm ruler as a scale for a later processing with Image J. For the calculation of the area contraction, the following equation was used:

$$SR_A=A_i/A_0$$

where SR_A is the area swelling ratio, A_i is the area of the hydrogel at each time point and A_0 is the area of the hydrogel at time zero.

The weight contraction of the hydrogels was evaluated by mass loss.[4] PLASMA_0, PLASMA_0.1 and PLASMA_0.2 (2 mL of final volume) were prepared in glass vials. Once the gelation reaction had taken place, the hydrogels were detached using 1 mL of warned PBS at 37ºC and transferred to pre-weighted p35 Petri dishes. The hydrogels were submerged in 3 mL of PBS (replenished after each measurement) and incubated at 37°C, 5% CO_2 and 40% relative humidity. Weight contraction was measured at time points of 0h, 1h, 3h, 6h, 24h, 48h and 7 days. The hydrogels were weighted in a precision balance. For the calculation of the weight loss, the following equation was used:

$$SR_W=M_i/M_0$$

where SR_W is the mass swelling ratio, M_i is the mass of the hydrogel at each time point and M_0 is the mass of the hydrogel at time zero.

1.2.3. Compression test:

In order to characterize the mechanical properties of the plasma-derived fibrin hydrogels, a DMA-Q800 equipment was used. PLASMA_0, PLASMA_0.1 and PLASMA_0.2 (2 mL of final volume) were prepared, and the mechanical measurements were performed at two different states: (i) freshly made hydrogels and (ii) hydrogels that had been incubated at 37°C inside glass vials and immersed in 3 mL of PBS for 24h. The hydrogels were subjected to a uniaxial and constant compression force of 0.1 N/min at a temperature of 37°C. The results of the applied force overtime gave information about stress-strain curves, and the compression moduli were obtained from the slope of the linear parts.[12] Before each measurement, the hydrogels were separated from the glass vials and the excess of liquid was soaked up with filter paper.

1.2.4. Morphological characterization by scanning electron microscopy (SEM)

The possible alterations on the structure morphologies of the hydrogels, not only in the freshly made ones, but also in the scaffolds that had been incubated in PBS for 3 days, and in the presence or not of GO, were characterized by Cryo-SEM, using a GeminiSEM 500 from Zeiss coupled with a Quorum cryo-SEM, under high vacuum conditions and an accelerated voltage of 2kV.

Additionally, cryo-FIB-SEM was performed using a Zeiss Crossbeam550 equipped with a Leica cryo-stage. The samples were prepared by direct gelation of the hydrogels (4 µL pre-gel solutions) into 200 µm deep planchettes for further vitrification process using a High Pressure Freezer Leica EM ICE machine.

1.3. Synthesis and characterization of GO/STREP

A mixture containing the drug and the nanomaterial was prepared from stock solutions of streptomycin (21.5 mg/mL) and GO (7.5 mg/mL), resulting in final concentrations of 16.73 mg/mL and 1.67 mg/mL, respectively. Ultrapure water was used as solvent and the mixture was stirred at 4°C overnight.

In order to measure the drug release of the hybrid, 0.5 mL of the just synthesized sample were centrifuged at 5000 rpms and 4°C for 5 min. Afterwards, the supernatant containing the unadsorbed drug was removed, stored and replaced with 0.5 mL of fresh ultrapure water. 200 µL of each supernatant was filtered with a 0.2 um pore size membrane in order to remove possible GO traces, and the drug content was evaluated by UV using a SynergyTM HTX Multi-Mode Microplate Reader (Winooski, VT, USA), measuring at 328 nm, and using a standard curve of different known concentrations of the drug. The washing process was repeated until no signal from streptomycin was observed.

The amount of drug loaded on the GO was estimated using the following equation:

Loading content (%) = [(initial mass of streptomycin − mass released in supernatant) / initial mass of streptomycin] ×100

Raman spectroscopy: at least 30 different measurements were collected, for each Raman spectrum average, in different locations of the samples (previously deposited on silicon wafer) using a Renishaw inVia Reflex Microscope at 532 nm with a 100× objective and an incident power of 1% (1 mW μm^{-2}).

Z-potential: the Z-potential of both GO and GO/STREP dispersions in MilliQ water (0.2 mg/mL) was measured at 25°C using a BIC 90Plus analyzer.

Thermogravimetric analysis (TGA): TGA curves of the freeze-dried dispersions of GO, STREP and GO/STREP materials were acquired by using a TGA Q50 instrument (TA Instruments Company) from 30 to 900°C with a ramp of 10 °C min^{-1} under N_2 using a flow rate of 50 mL min^{-1} and platinum pans.

1.4. Bactericidal studies

In order to quantify the bactericidal ability of our hydrogels containing (or not) the GO/STREP hybrid, we performed the spread plate (or colony counting) method using *Escherichia coli (E. coli)*.[13,14]

Firstly, *E. coli* cells were growth in Lysogeny Broth (LB) medium at 37°C under 210 rpm shaking speed and turbidity was adjusted to 1.9e5 CFU/mL (O.D. was measured with a Biowave II spectrophotometer (Biochrom, UK) at 600 nm). The cells were harvested by centrifugation, then washed twice with PBS and resuspended in the appropriate saline medium. *E. coli* were incubated with the different freshly prepared hydrogel samples (pre-gels volumes of 300 µL) in PBS at 37°C under 210 rpm shaking speed for 1h or 2h. Aliquots of samples were withdrawn, diluted and then spread onto LB agar plates. After incubation at 37°C, the capacity of the bacteria to form colonies was measured by counting the number of colony-forming units. All the treatments were performed at least in triplicate.

1.5. Cytotoxicity studies

1.5.1. LDH assay:

LDH is a soluble but stable enzyme present inside every living cell. When the cell membranes are compromised, this enzyme is released into the surrounding extracellular space. LDH is an oxidoreductase which catalyzes the interconversion of lactate and pyruvate, which allows to detect the leakage of this enzyme into the cell culture medium using a tetrazolium salt that is converted into a highly colored formazan product which can be quantified by standard spectroscopy.[15] This cytotoxic assay was performed with both nanomaterials (i.e. GO/STREP dispersions) and hydrogels (i.e. PLASMA_0, PLASMA_0.1_GO/STREP and PLASMA_0.2_GO/STREP) at least in triplicates.

hFBs were seeded in a 96 microwell plate (5000 cells/well). After allowing the cells to attach to the plastic surface, both the nanomaterials and the just prepared hydrogels were added to the wells and placed in contact with the cells for 24h. Positive control consisted of the same concentration of hFBs without any treatment. DMSO (10%), was also used as the negative control since it is toxic for the cells. Then, a Cytotoxic 96@ Non-Radioactive kit from Promega was used as recommended. Firstly, of 15 µL of lysis buffer were added per well. After incubating for 50

min at 37°C, 5% CO_2 and 90% relative humidity, the supernatants were centrifuged to remove any trace of nanomaterial that could cause interferences in the absorbance. Afterwards, 50 µL of each supernatant were mixed with 50 µL of Cytotoxic 96 Reagent for 10 min and covered with aluminium foil and finally, 50 µL of stop solution was added to the mix. As a last step, any air bubble was removed, and the values of absorbance were read with a SynergyTM HTX Multi-Mode Microplate Reader (Winooski, VT, USA).

1.5.2. Live/Dead® assay:

Cell viability of hFBs was also characterized using Live/Dead® assay for mammalian cells. This method is based on the simultaneous detection of live and dead cells by the recognition of specific molecular probes. Death cell detection was determined by EthD-1 that can enter through damaged cell membranes of non-viable cells and bind to nucleus which induces a bright red fluorescence expression (ex/em ~495 nm/~635 nm). In live cells, the presence of intracellular esterase converts cell-permeant Calcein AM into green, fluorescent Calcein (ex/em ~495 nm/~515 nm).[16]

hFBs were seeded in a 96 microwell plate (5000 cells/well). After allowing the cells to attach to the plastic surface, the just prepared hydrogels were added to the wells and placed in contact with the cells for 24h. Positive control consisted of the same concentration of hFBs without any treatment. DMSO (10%), was also used as the negative control since it is toxic for the cells. Culture plate was taken off the incubator and culture media were carefully removed with a micropipette. hFBs were washed twice for 5 minutes using a nutator at medium speed. Meanwhile, Live/Dead® working solution was prepared in PBS 1X by the addition of Calcein-AM and EthD-1 in a final concentration of 2µM. After washing, PBS 1X was discarded and 50 µL of working solution was added to each well and incubated in darkness at room temperature for 45 minutes. Finally, hFBs were washed twice with PBS 1X for 15 minutes in a nutator at medium speed to avoid background signal. Fluorescence images were visualized using inverted microscope Leica Dmi8.

Bibliography

(1) Morimoto, N.; Kubo, T.; Nishina, Y. Tailoring the Oxygen Content of Graphite and Reduced Graphene Oxide for Specific Applications. *Sci. Reports 2016 61* **2016**, *6* (1), 1–8. https://doi.org/10.1038/srep21715.

(2) Kinoshita, H.; Nishina, Y.; Alias, A. A.; Fujii, M. Tribological Properties of Monolayer Graphene Oxide Sheets as Water-Based Lubricant Additives. *Carbon* **2014**, *66*, 720–723. https://doi.org/10.1016/J.CARBON.2013.08.045.

(3) Morimoto, N.; Suzuki, H.; Takeuchi, Y.; Kawaguchi, S.; Kunisu, M.; Bielawski, C. W.; Nishina, Y. Real-Time, in Situ Monitoring of the Oxidation of Graphite: Lessons Learned. *Chem. Mater.* **2017**, *29* (5), 2150–2156. https://doi.org/10.1021/ACS.CHEMMATER.6B04807/SUPPL_FILE/CM6B04807_SI_001.PDF.

(4) Montero, A.; Acosta, S.; Hernández, R.; Elvira, C.; Jorcano, J. L.; Velasco, D. Contraction of Fibrin-Derived Matrices and Its Implications for in Vitro Human Skin Bioengineering. *J. Biomed. Mater. Res. Part A* **2021**, *109* (4), 500–514. https://doi.org/10.1002/JBM.A.37033.

(5) Cook, M. T.; Haddow, P.; Kirton, S. B.; McAuley, W. J. Polymers Exhibiting Lower Critical Solution Temperatures as a Route to Thermoreversible Gelators for Healthcare. *Adv. Funct. Mater.* **2021**, *31* (8). https://doi.org/10.1002/adfm.202008123.

(6) Weigandt, K. M.; White, N.; Chung, D.; Ellingson, E.; Wang, Y.; Fu, X.; Pozzo, D. C. Fibrin Clot Structure and Mechanics Associated with Specific Oxidation ofMethionine Residues in Fibrinogen. *Biophys. J.* **2012**, *103* (11), 2399. https://doi.org/10.1016/J.BPJ.2012.10.036.

(7) Lee, F.; Kurisawa, M. Formation and Stability of Interpenetrating Polymer Network Hydrogels Consisting of Fibrin and Hyaluronic Acid for Tissue Engineering. *Acta Biomater.* **2013**, *9* (2), 5143–5152. https://doi.org/10.1016/j.actbio.2012.08.036.

(8) Sanz-Horta, R.; Matesanz, A.; Jorcano, J. L.; Velasco, D.; Acedo, P.; Gallardo, A.; Reinecke, H.; Elvira, C. Preparation and Characterization of Plasma-Derived Fibrin Hydrogels Modified by Alginate Di-Aldehyde. *Int. J. Mol. Sci.* **2022**, *23* (8), 4296. https://doi.org/10.3390/IJMS23084296.

(9) Mao, B.; Divoux, T.; Snabre, P. Normal Force Controlled Rheology Applied to Agar Gelation. *J.*

Rheol. **2016**, *60* (3), 473. https://doi.org/10.1122/1.4944994.

(10) Dou, Q.; Karim, A. A.; Loh, X. J. Modification of Thermal and Mechanical Properties of PEG-PPG-PEG Copolymer (F127) with MA-POSS. *Polym.* **2016**, *8* (9), 341. https://doi.org/10.3390/POLYM8090341.

(11) Rittié, L.; Berton, A.; Monboisse, J. C.; Hornebeck, W.; Gillery, P. Decreased Contraction of Glycated Collagen Lattices Coincides with Impaired Matrix Metalloproteinase Production. *Biochem. Biophys. Res. Commun.* **1999**, *264* (2), 488–492. https://doi.org/10.1006/BBRC.1999.1519.

(12) Stojic, M.; Ródenas-Rochina, J.; López-Donaire, M. L.; de Torre, I. G.; Pérez, M. G.; Rodríguez-Cabello, J. C.; Vojtová, L.; Jorcano, J. L.; Velasco, D. Elastin-Plasma Hybrid Hydrogels for Skin Tissue Engineering. **2021**, *13* (13), 2114. https://doi.org/10.3390/POLYM13132114.

(13) Yu, Z. H.; Li, X.; Xu, F.; Hu, X. Le; Yan, J.; Kwon, N.; Chen, G. R.; Tang, T.; Dong, X.; Mai, Y.; Chen, D.; Yoon, J.; He, X. P.; Tian, H. A Supramolecular-Based Dual-Wavelength Phototherapeutic Agent with Broad-Spectrum Antimicrobial Activity Against Drug-Resistant Bacteria. *Angew. Chemie - Int. Ed.* **2020**, *59* (9), 3658–3664. https://doi.org/10.1002/anie.201913506.

(14) Park, S. Y.; Kim, C. G. A Comparative Study of Three Different Viability Tests for Chemically or Thermally Inactivated Escherichia Coli. *Environ. Eng. Res.* **2018**, *23* (3), 282–287. https://doi.org/10.4491/eer.2017.223.

(15) CytoTox 96® Non-Radioactive Cytotoxicity Assay Technical Bulletin phttps://www.promega.es/resources/protocols/technical-bulletins/0/cytotox-96-non-radioactive-cytotoxicity-assay-protocol/ (accessed March 10, 2023).

(16) LIVE/DEAD ® Viability/Cytotoxicity Kit *for Mammalian Cells Https://Www.Thermofisher.Com/Document-Connect/Document-Connect.Html?Url=https://Assets.Thermofisher.Com/TFS-Assets%2FLSG%2Fmanuals%2Fmp03224.Pdf (Accessed March 10, 2023).

Supplementary Figures

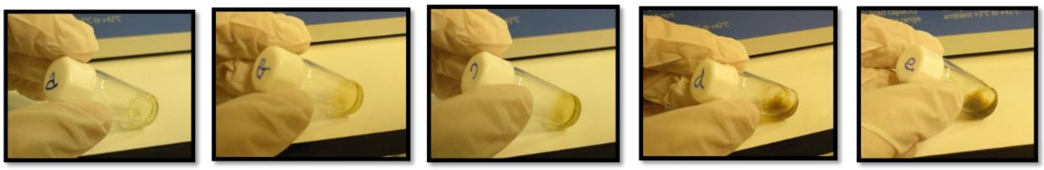

PLASMA_0 PLASMA_0.1 PLASMA_0.2 PLASMA_0.3 PLASMA_0.5

Figure S1. Digital pictures of gel mixtures containing from 0 mg/mL to 0.5 mg/mL of GO after 1h at 37°C. A homogeneous one-piece hydrogel is obtained up to 0.2 mg/mL of GO (PLASMA_0.2). A “heterogeneous dispersion” consisting of many hydrogel particles are obtained above 0.2 mg/mL.

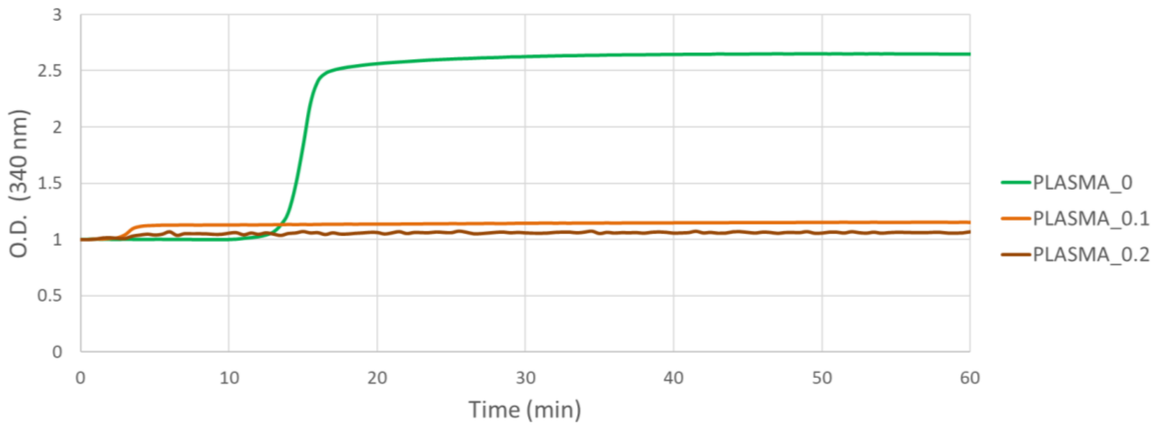

Figure S2. Representative gelation kinetics of PLASMA_0, PLASMA_0.1 and PLASMA_0.5 by optical density at 430 nm. The experiments were performed in triplicates, but one sample of each concentration is represented for simplification due to the good reproducibility.

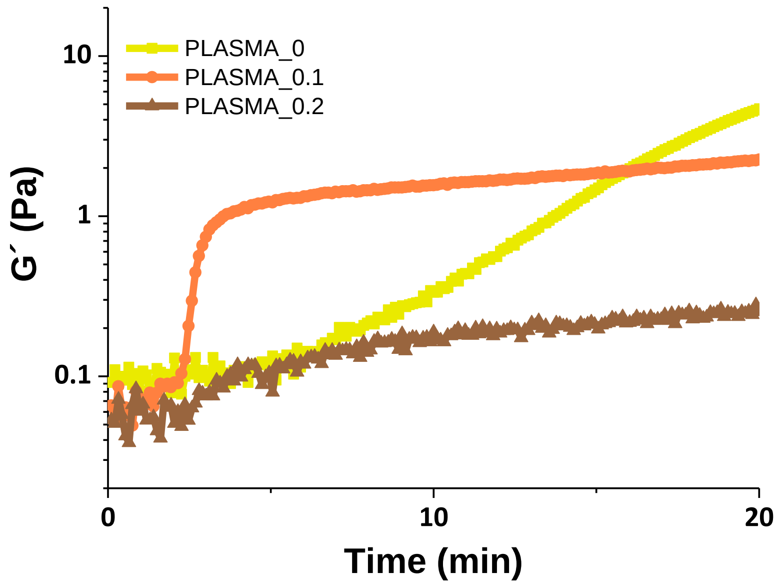

Figure S3. Representative gelation kinetics behaviors via rheological analysis for PLASMA_0, PLASMA_0.1 and PLASMA_0.2, in a log scale. The experiments were performed in triplicates, but one sample of each hydrogel is represented for simplification.

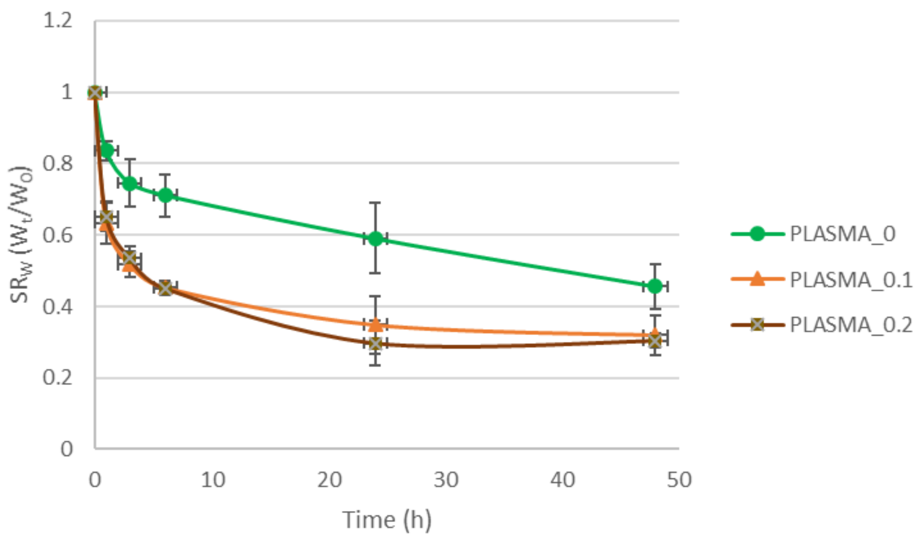

Figure S4. Weight loss profiles for PLASMA_0, PLASMA_0.1 and PLASMA_0.2. Data for time 7 days are not shown for clarification.

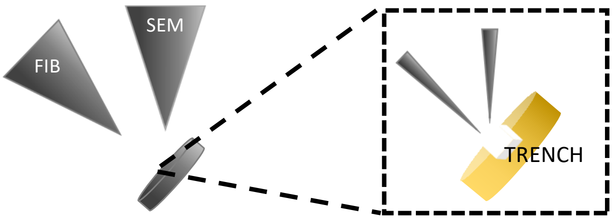

Figure S5. Schematic representation of the cryo-FIB-SEM technique.

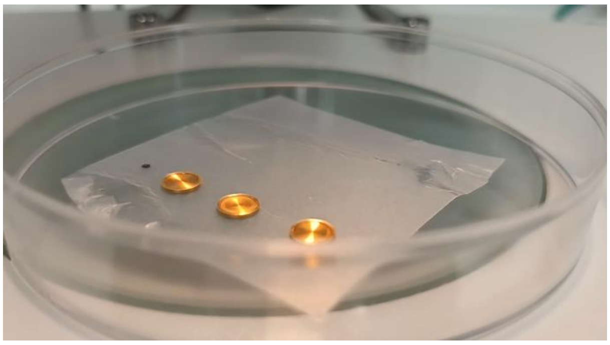

Figure S6. Digital picture of 200 µm deep planchettes containing 4µL of the pre-gel solutions.

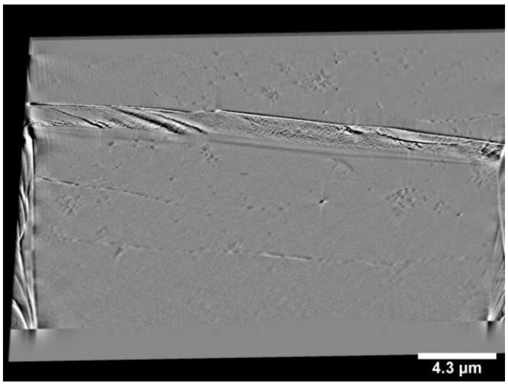

Figure S7. Cryo-FIB-SEM image of the pre-gel solution of PLASMA_0. No fibers can be clearly identified since the pre-gel is vitrified at t=0, before fibrin polymerization starts. Scale bar: 4.3 µm.

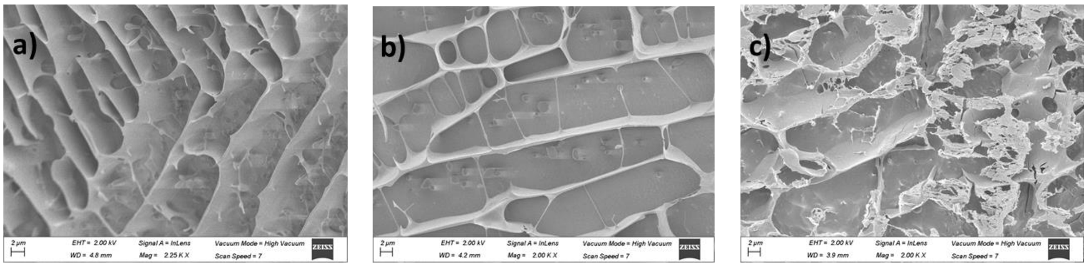

Figure S8. Representative Cryo-SEM images of the pore-size and morphologies corresponding to the freshly made materials a) PLASMA_0, b) PLASMA_0.1 and c) PLASMA_0.2. Scale bar: 2 µm.

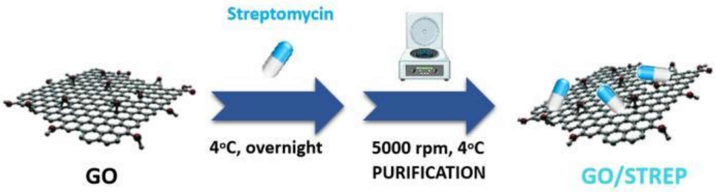

Figure S9. Scheme of the synthesis and purification steps of the GO/STREP hybrid.

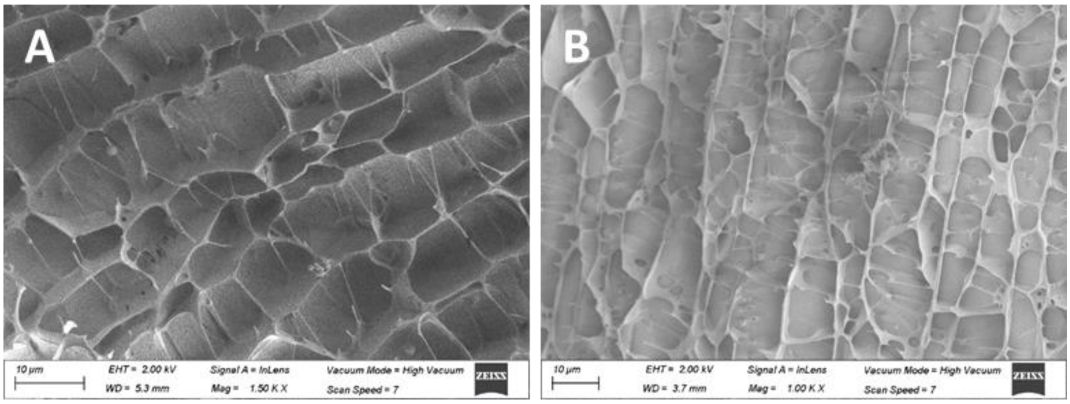

Figure S10. Representative cryo-SEM images of freshly made (A) PLASMA_0.1_GO/STREP and (B) PLASMA_0.2_GO/STREP hydrogels (scale bars: 10 µm).

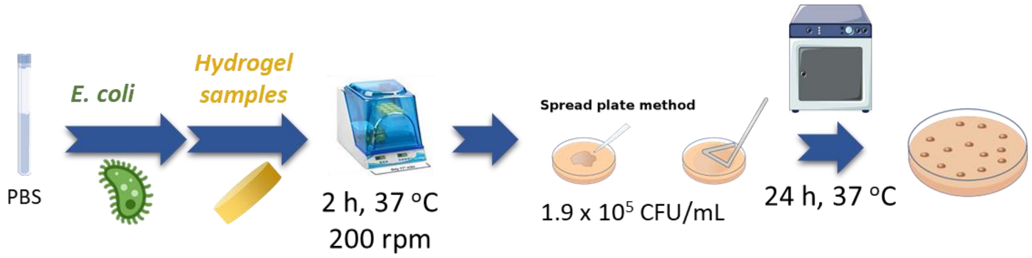

Figure S11. Schematic representation of the protocol followed to perform the bactericidal studes.

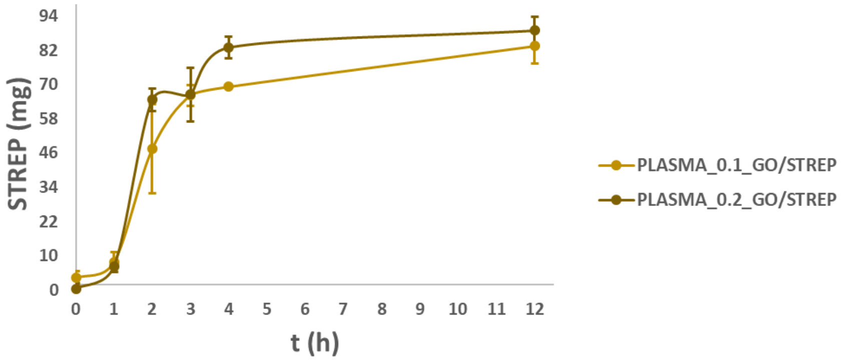

Figure S12. Streptomycin release kinetics for PLASMA_0.1_GO/STREP and PLASMA_0.2_GO/STREP hydrogels.

1 h of incubation	2 h of incubation
A-PLASMA_0 (CONTROL)	B-PLASMA_0 (CONTROL)
C-PLASMA_0.1_GO/STREP	D-PLASMA_0.1_GO/STREP
E-PLASMA_0.2_GO/STREP	F-PLASMA_0.2_GO/STREP

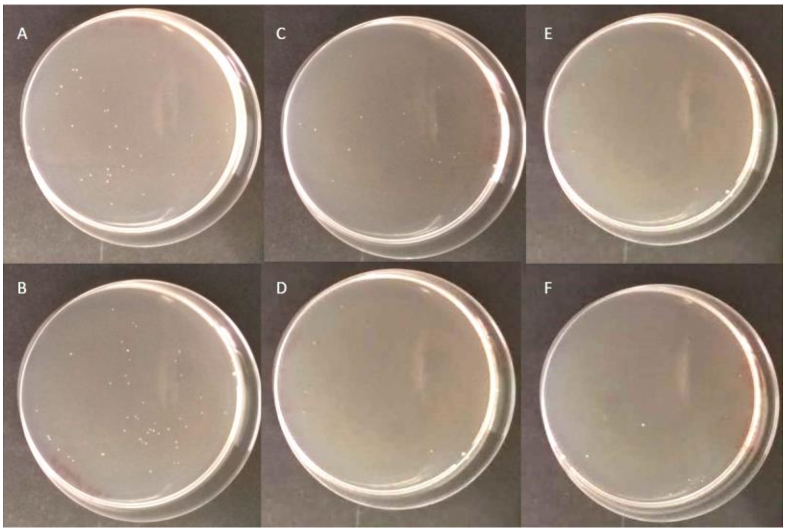

Figure S13. Letter-sample correlations and representative corresponding agar plates of *E. coli* colonies that resulted from the different hydrogel samples tested against bacteria after 1h (A, C, E) and 2h (B, D, F) of incubation.

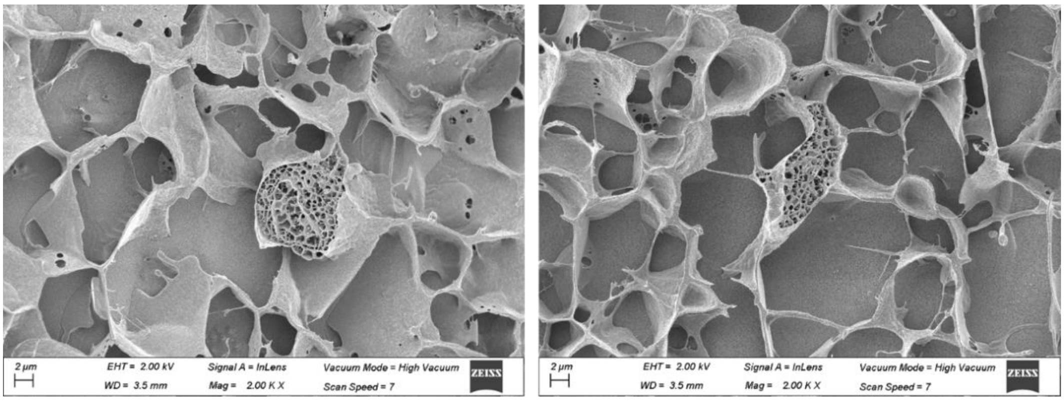

Figure S14. Representative cryo-SEM image of hFBs embedded into PLASMA_0.2_GO/STREP hydrogel (scale bars: 2 µm).

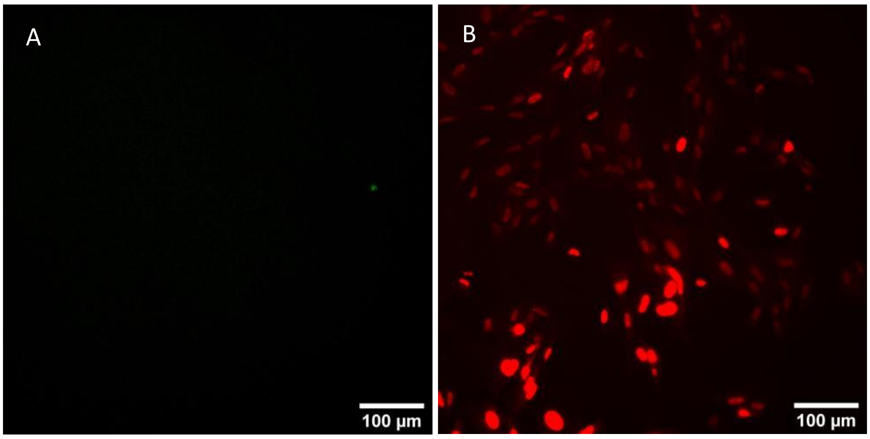

Figure S15. Representative images obtained from the Live/Dead experiment carried out with the negative control sample, which consisted of hFBs that were exposed to 10%DMSO for 24h.
